# An engineered oncolytic virus expressing PD-L1 inhibitors activates tumor neoantigen-specific T cell responses

**DOI:** 10.1038/s41467-020-15229-5

**Published:** 2020-03-13

**Authors:** Guan Wang, Xi Kang, Katherine S. Chen, Tiffany Jehng, Lindsey Jones, Jie Chen, Xue F. Huang, Si-Yi Chen

**Affiliations:** 1 Department of Molecular Microbiology and Immunology, Norris Comprehensive Cancer Center, Keck School of Medicine, University of Southern California, Los Angeles, 90033 CA USA; 2 Fu Foundation School of Engineering and Applied Science, Columbia University, New York, 10023 NY USA; 3 Pomona Biotechnology Corp., Beijing, 100176 China; 4 LifeSeq Limited Corp, Monrovia, 91016 CA USA

**Keywords:** Cancer immunotherapy, Immunology, Cancer immunotherapy

## Abstract

Oncolytic viruses offer an in situ vaccination approach to activate tumor-specific T cell responses. However, the upregulation of PD-L1 expression on tumor cells and immune cells leads to tumor resistance to oncolytic immunotherapy. In this study, we generate an engineered oncolytic virus that coexpresses a PD-L1 inhibitor and GM-CSF. We find that the oncolytic virus is able to secrete the PD-L1 inhibitor that systemically binds and inhibits PD-L1 on tumor cells and immune cells. Importantly, the intratumoral injection with the oncolytic virus overcomes PD-L1-mediated immunosuppression during both the priming and effector phases, provokes systemic T cell responses against dominant and subdominant neoantigen epitopes derived from mutations, and leads to an effective rejection of both virus-injected and distant tumors. In summary, this engineered oncolytic virus is able to activate tumor neoantigen-specific T cell responses, providing a potent, individual tumor-specific oncolytic immunotherapy for cancer patients, especially those resistant to PD-1/PD-L1 blockade therapy.

## Abstract (web summary)

Oncolytic viruses can activate tumor neoantigen-specific T cell responses. However, PD-L1 upregulation on tumor cells and immune cells leads to tumor resistance to this immunotherapy approach. Here, the authors develop an oncolytic virus which expresses both a PD-L1 inhibitor and GM-CSF which allows an improved tumor neoantigen-specific T cell response in preclinical models.

## Introduction

Cancer is a genetic disease, with the growth of tumor cells initiated by mutations that activate oncogenic drivers and disable tumor suppressors. Recent studies have demonstrated that tumor neoantigens can be derived de novo from the expression of genetic mutations and presented in major histocompatibility complexes (MHC) on tumor cells, and endogenous T cell responses against neoantigens can be naturally activated in cancer patients^1–4^. However, only a small number of nonsynonymous mutations expressed in tumors can be adequately presented as neoantigens for which the T cell response can be mounted^5,6^.

Compounding this problem of inefficient neoantigen presentation is the immunosuppressive tumor microenvironment that inhibits antitumor T cell responses by immune checkpoint molecules, such as PD-1/PD-L1^7,8^. Immune checkpoint blockade effectively augments endogenous T cell responses against tumor neoantigens and led to the enduring responses in patients with advanced malignancies, including complete responses in various types of cancer, such as melanoma, metastatic lung, kidney, and bladder carcinoma^7,8^. Responses to PD-1 inhibition are highly correlated with the presence of CD8^+^ T cells at the invasive margin and within the tumor lesions, which define the so-called inflamed “hot” tumors^9^. However, the majority of cancer patients are resistant to PD-1/PD-L1 blockade. One reason for treatment failures is attributed to the so-called “cold” tumors, which might have low mutational burden and neoantigen load, poor MHC presentation, and poor capacity to attract T cell infiltration^10–15^.

Increasing the response rates to PD-1 blockade therapy remains an important challenge, given that the majority of tumors fail to spontaneously provoke T cell responses against tumor mutant neoantigens and are resistant to PD-1 blockade. Recently, intensive efforts have been devoted to activating neoantigen-specific T cell responses^16^. Neoantigen-specific T cells can be activated by comprehensive sequencing and the identification of individual mutations, the computational prediction of neoantigen epitopes, and vaccination with neoantigen epitopes for each patient^16–18^. In contrast to this cumbersome strategy, oncolytic viruses possess the potential to offer a simpler in situ vaccination approach to activate T cell responses by locoregional immune activation, immunogenic oncolytic tumor cell death, mutant neoantigen release and presentation, and alteration of the immunosuppressive tumor microenvironment^19,20^. Recent clinical trials demonstrated that oncolytic virotherapy with talimogene laherparepvec (T-Vec), a genetically modified granulocyte-macrophage colony-stimulating factor (GM-CSF)-expressing herpes simplex virus, promoted intratumoral T cell infiltration and improved anti-PD-1 or CTLA immunotherapy^21,22^. However, how oncolytic viruses activate tumor neoantigen-specific T cell responses is still poorly studied. Moreover, the problem that the reactive upregulation of PD-L1 expression in the tumor microenvironment after virus administration can cause tumor resistance to oncolytic immunotherapy^23,24^ should be overcome. In this study, we generate an engineered oncolytic vaccinia virus ((VV)-iPDL1/GM) that coexpresses a PD-L1 inhibitor and GM-CSF. We find that this engineered oncolytic virus is capable of activating neoantigen-specific T cell responses by the likely synergistic action of viral replication, GM-CSF stimulation, and PD-L1 inhibition on tumor cells and immune cells, providing a novel oncolytic immunotherapy.

## Results

### Generation and characterization of an armed oncolytic VV coexpressing a PD-L1 inhibitor and GM-CSF (VV-iPDL1/GM)

**Fig. 1:**
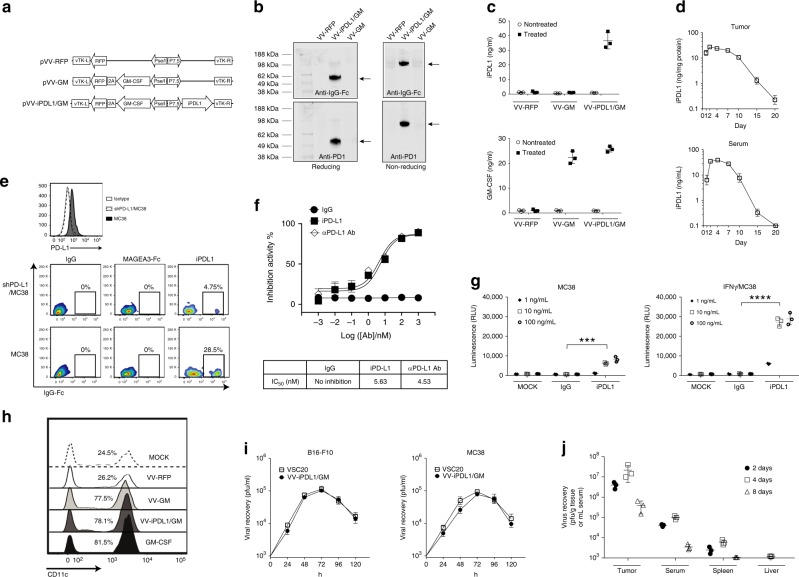
Generation and characterization of an oncolytic vaccinia virus coexpressing a mouse PD-L1 inhibitor and GM-CSF. **a** A schematic diagram of recombinant vaccinia virus (VV) shuttle vectors that express GM-CSF or/and iPDL1 (soluble PD-1-Fc). vTK, VV thymidine kinase gene; R and L, right and left flank sequences; RFP, red fluorescent protein. **b** Expression and secretion of iPDL1 from infected MC38 tumor cells infected with the indicated VVs. Anti-IgG Fc (Licor 926-32210; upper) or anti-PD-1 (Biolegend 114101; lower) was used for western blot with reducing or non-reducing loading buffer. The experiment was repeated twice. **c**, **d** Serum iPDL1 and GM-CSF levels in different VV-treated MC38-bearing mice at 2 days post-virus injection **c**. Kinetics of iPDL1 levels in injected tumors or sera of the VV-iPDL1/GM-treated mice **d**. *n* = 3 independent samples. Data are presented as the means ± SD. The experiment was repeated twice. **e** Purified iPDL1 binds to PD-L1^+^ tumor cell. Upper panel: flow cytometric analysis of PD-L1 expression on shPD-L1/MC38 tumor cells that were transduced with PD-L1-shRNA and wild-type MC38 cells. Lower panel: shPD-L1/MC38 cells and wild-type MC38 cells were incubated with 50 μg/mL of purified iPDL1, an irrelevant MAGE3-IgG Fc fusion protein, or IgG control, followed by staining with an anti-IgG Fc for flow cytometry. **f** Inhibition of PD-1/PD-L1 binding by purified iPDL1 protein using ELISA. An anti-PD-L1 antibody was used as a positive control; *n* = 3 independent samples. **g** iPDL1-mediated ADCC. ADCC Reporter Bioassays were performed in triplicate wells, and the concentrations of iPDL1 protein and control IgG Fc used for this assay are indicated; *n* = 3 independent samples. Data presented as the means ± SD. The experiment was repeated twice. Significant differences are indicated as ***P < 0.001, or ****P < 0.0001 using two-tailed student’s *t*-test. **h** CD11c^+^ DC frequency in monocyte cultures in the presence of culture media of MC38 cells infected with VV-RFP, VV-GM, VV-iPDL1/GM, or GM-CSF as a positive control, and IL-4. **i** Viral replication in vitro; *n* = 3 independent samples. **j** Replication and biodistribution of VV after intratumor injections. Data presented as the means ± SD. The experiment was repeated twice.

We generated an engineered oncolytic VV coexpressing a murine soluble PD-1 extracellular domain fused with IgG1 Fc as a PD-L1 inhibitor (i.e., iPDL1) and murine GM-CSF (VV-iPDL1/GM), in the backbone of a tumor-selective double-deleted oncolytic VV, in which thymidine kinase (TK) and vaccinia growth factor viral genes had been deleted^25–29^ (Fig. 1a). A recombinant oncolytic VV-GM expressing murine GM-CSF and a recombinant oncolytic VV-RFP expressing the marker RFP were also generated and produced. High levels of both GM-CSF and iPDL1 (soluble PD-1-IgG Fc) proteins in a dimer were produced and efficiently released from VV-iPDL1/GM-infected tumor cells in vitro and in vivo, as detected by western blot and enzyme-linked immunosorbent assay (ELISA; Fig. 1b–d, Supplementary Fig. 1). Importantly, high levels of iPDL1 were detected in the sera of VV-iPDL1/GM-treated tumor-bearing mice for a long period of time (over 15 days) after intratumor injection (Fig. 1d).

iPDL1 protein purified from the supernatants of VV-iPDL1/GM-infected tumor cells^30,31^ was able to bind to PD-L1^+^ tumor cells, but not to PD-L1-knocked down tumor cells in vitro (Fig. 1e). In addition, it was shown that MAGEA3-IgG Fc fusion proteins failed to bind to PD-L1^+^ tumor cells, further ruling out the non-specific binding of IgG Fc domain to tumor cells. iPDL1 had a comparable IC_50_ value with the commercial anti-PD-L1 antibody in blocking PD-1/PD-L1 interaction, as manifested by a competitive ELISA assay (Fig. 1f). It was found that iPDL1, but not IgG1 Fc, efficiently mediated antibody-dependent cell-mediated cytotoxicity (ADCC) against IFNγ-treated, PD-L1-expressing tumor cells (Fig. 1g). Supernatants derived from VV-GM- or VV-iPDL1/GM-infected MC38 tumor cells also had GM-CSF functionality in driving bone marrow (BM)-derived monocytes to differentiate into CD11c^+^ DCs (Fig. 1h). Moreover, the insertion of iPDL1 gene into the oncolytic VV did not interfere with the infection and replication of VV-iPDL1/GM in vitro and in vivo (Fig. 1i, j, Supplementary Fig. 2). Taken together, these data demonstrate that the armed oncolytic virus VV- iPDL1/GM can infect tumor cells to produce and secrete high levels of functional iPDL1 and GM-CSF proteins.

### PD-L1 inhibitors secreted from VV-iPDL1/GM-infected cells bind to upregulated PD-L1 on tumor cells and immune cells in autocrine and paracrine manners

**Fig. 2:**
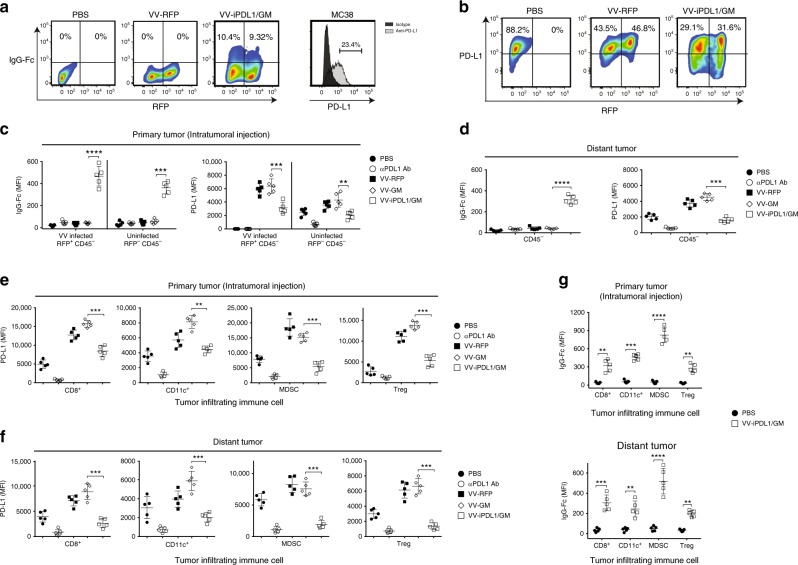
PD-L1 inhibitors secreted from VV-iPDL1/GM-infected cells bind to PD-L1 on tumor cells and immune cells. **a** MC38 tumor cells were infected with VV-RFP, VV-iPDL1/GM at an MOI = 0.5, or PBS for 24 h. The percentage of IgG Fc^+^ population representing iPDL1 (soluble PD-1-IgG Fc)-bound VV-infected (RFP^+^) or uninfected (RFP^−^) PD-L1-expressing tumor cells was measured by flow cytometry. **b** MC38 tumor cells that were stimulated with IFN-γ (20 ng/mL) for 48 h were infected with the indicated VVs. PD-L1 expression of infected (RFP^+^) or uninfected (RFP^−^) cells was determined by flow cytometry. **c**–**g** MC38 cells were subcutaneously inoculated into the left (1 × 10^6^) and right (5 × 10^5^) flanks of C57BL/6 mice. When left flank tumor sizes reached ~100 mm^3^ (counted as day 0), the tumors of the left flank were intratumorally injected with 50 μL of PBS, VV-RFP, VV-GM, or VV-iPDL1/GM (5 × 10^7^ pfu per tumor), or 200 μg of anti-PD-L1 antibody (clone 10F.9G2) intravenously on days 0 and 3. Two days post-second VV treatment, VV-treated (primary tumor) and untreated, distant tumors were collected, weighed and digested with collagenase type I and DNase. Tumor cell suspensions were blocked with anti-CD16/32 antibody and then stained with antibodies against CD45, CD3, CD8, CD4, CD11c, CD11b, Gr-1, FoxP3, PD-L1, and IgG Fc to assess PD-L1 expression or IgG Fc^+^ frequency on infected (RFP^+^) or uninfected (RFP^−^) tumor cells from the treated primary tumors **c** or untreated distant tumors (CD45^−^ cells) **d**, and PD-L1 expression on infiltrating immune cells from treated **e** or untreated distant tumors **f**, IgG Fc^+^ frequency on infiltrating immune cells **g**. Infiltrating immune cells include cytotoxic T cells (CD45^+^CD3^+^CD8^+^), DCs (CD45^+^CD11c^+^), myeloid-derived suppressor cell MDSCs (CD45^+^CD11c^−^CD11b^+^Gr-1^+^), and Treg (CD45^+^CD3^+^CD4^+^FoxP3^+^); *n* = 5 mice. Significant differences are indicated as **P < 0.01, ***P < 0.001, or ****P < 0.0001 determined by two-tailed Student’s *t*-test.

We examined whether iPDL1 secreted from VV- iPDL1/GM-infected tumor cells was able to bind PD-L1 on tumor cells in cell culture. Figure 2a shows that the secreted iPDL1 (PD-1-IgG Fc) bound to PD-L1 on the virus-infected (RFP positive) tumor cells, as well as uninfected (RFP negative) tumor cells in autocrine and paracrine manners by flow cytometry staining with an anti-IgG Fc to detect the binding of secreted iPDL1 to PD-L1 on tumor cells. The percentage of PD-L1^+^ VV-iPDL1/GM-infected (RFP^+^) or uninfected (RFP^−^) tumor cells was significantly lower than that of VV-RFP-infected (RFP^+^) or uninfected (RFP^−^) tumor cells (Fig. 2b), suggesting that the binding of the iPDL1 secreted from VV-iPDL1/GM-infected cells to PD-L1 on tumor cells partially blocked PD-L1 staining with an PD-L1 antibody.

We then examined whether VV-iPDL1/GM-infected cells were able to secrete iPDL1 that can bind PD-L1 on tumor cells in vivo. Groups of mice bearing MC38 tumors in right and left flanks were injected with the recombinant VV-iPDL1/GM, VV-GM, or VV-RFP only into the tumors in the left flank. Single-cell suspensions prepared from treated primary tumors or untreated, distant tumors were analyzed by flow cytometry staining with an anti-IgG Fc. Consistent with the observations in recent studies^23,24^, intratumoral injections with oncolytic viruses (VV-RFP) also significantly upregulated PD-L1 expression on both VV-RFP-infected (RFP^+^) and uninfected (RFP^−^) CD45^−^ non-leukocyte cells, including tumor and stromal cells, compared to PD-L1 expression on PBS-treated tumors (Fig. 2c). Lower levels of PD-L1 expression in the tumors injected with VV-iPDL1/GM, compared to VV-RFP, suggesting that the secreted iPDL1 bound to PD-L1 (Fig. 2c). Indeed, the binding of secreted iPDL1 (PD-1-IgG Fc) to PD-L1 on VV-iPDL1/GM-treated CD45^−^ non-leukocyte cells was detected (Fig. 2c). Importantly, iPDL1 (PD-1-IgG Fc) secreted from treated primary tumors also bound to PD-L1 on CD45^−^ cells in untreated, distant tumors (Fig. 2d). These data indicate that the secreted iPDL1 binds to PD-L1 on CD45^−^ tumor and stromal cells in VV-iPDL1/GM-treated primary and untreated, distant tumors in autocrine and paracrine manners.

We further examined whether the iPDL1 secreted from VV-iPDL1/GM-treated tumors was able to bind PD-L1 on immune cells in vivo. The upregulation of PD-L1 expression on CD45^+^ hematopoietic cell infiltrates, including DCs, MDSCs, and T cells, was observed in both VV-RFP-treated and untreated tumors, compared to PD-L1 expression in PBS-treated tumors (Fig. 2e, f). Lower levels of PD-L1 expression on CD45^+^ hematopoietic cell infiltrates in VV-iPDL1/GM-injected tumors and distant tumors were detected compared to the PD-L1 expression in VV-RFP or VV-GM-injected tumors and distant tumors (Fig. 2e, f). Figure 2g shows the binding of secreted iPDL1 to PD-L1 on immune cells from VV-iPDL1/GM-treated and untreated distant tumors. Furthermore, we investigated the infection and secretion of iPDL1 after intratumor injections of VV-iPDL1/GM in tumor-bearing mice (Supplementary Fig. 3). Supplementary Figs. 4–6 show the efficient infection (RFP^+^) of tumor cells (CD45^−^CD31^−^Ter119^−^) by VV-iPDL1/GM in vivo. Supplementary Figs. 7 and 8 show the secretion of the iPDL1 dimer from the isolated tumor cells after intratumor injections with the binding activity to PD-L1 on immune cells. Taken together, these data demonstrate that iPDL1 secreted from VV-iPDL1/GM-treated tumors is able to systemically bind to PD-L1 on tumor cells and immune cells in vivo.

### Enhanced antitumor activities against primary and distant tumors

**Fig. 3:**
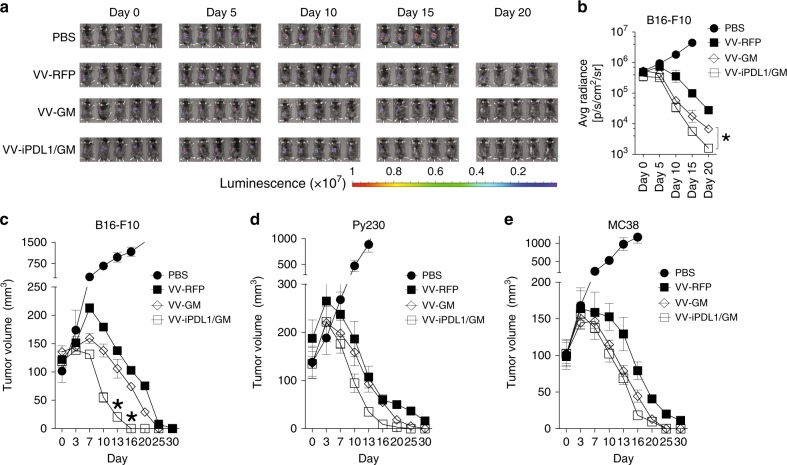
Enhanced antitumor activities against primary tumors. **a**–**c** C57BL/6 mice were subcutaneously inoculated with 5 × 10^5^ luciferase-expressing B16-F10 (B16-F10-Luc) cells. When tumor sizes reached ~100 mm^3^ (counted as day 0), the mice were intratumorally injected with 50 μL of VV-RFP, VV-GM, or VV-iPDL1/GM (5 × 10^7^ pfu per tumor) or PBS at days 0, 3, and 7. Bioluminescence monitoring **a**, **b** and caliper measurement **c** of B16-F10-Luc cells were performed on the indicated days. Data are presented as the means ± SD (*n* = 5 mice). Significant differences are indicated as *P < 0.05 determined by two-tailed Student’s *t*-test. **d**, **e** Py230 **d** or MC38 **e** tumor volume was monitored by caliper measurement using the same treatment schedule as in **a**–**c**. Data are presented as the means ± SD (*n* = 5 mice).

We evaluated the antitumor activity of VV-iPDL1/GM using a luciferase^+^ B16-F10 melanoma syngeneic transplant mouse model, which was weakly immunogenic. Tumor-bearing mice received intratumoral injections of various VVs or PBS as described in Methods section. Although intratumoral injections with VV-RFP or VV-GM drastically inhibited tumor growth, both bioluminescence monitoring (Fig. 3a, b) and caliper measurement (Fig. 3c) showed that VV-iPDL1/GM was more potent in inhibiting B16-F10 tumor growth. Intratumoral injections of the recombinant VVs also drastically inhibited the growth of Py230 breast cancer and MC38 colon adenocarcinoma (Fig. 3d, e).

**Fig. 4:**
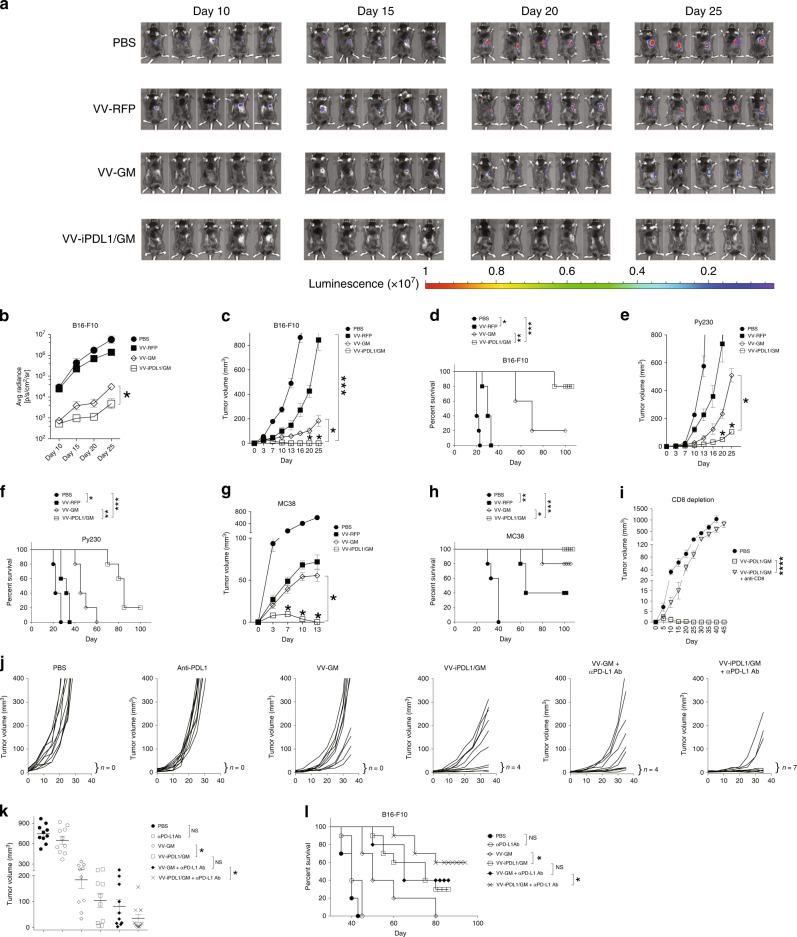
Enhanced antitumor activities against untreated, distant tumors. **a**–**d** Inhibition of rechallenged tumor growth. B16-F10 melanoma cells were implanted intradermally to the left flank of C57B/6 mice. When tumor sizes reached ~100 mm^3^ (counted as day 0), the mice were intratumorally injected with the indicated VVs on days 0, 3, and 7. Treated mice were s.c. rechallenged with B16-F10-Luc cells 30 days after the last VV injection (counted as day 0 for rechallenge data). Bioluminescence monitoring **a**, **b** and caliper measurement of B16-F10-Luc cells **c** were performed. Data are presented as means ± SD (*n* = 5 mice). **d** Survival curve of B16-F10 rechallenged mice. **e**–**h** The volumes of rechallenged Py230 **e** or MC38 **g** tumors were monitored using a similar treatment schedule as in **a**, except that 5 × 10^5^ of Py230 or MC38 tumor cells were rechallenged. Data are presented as means ± SD (*n* = 5 mice). *P < 0.05, ***P < 0.001 determined by two-way ANOVA. Survival curve of Py230 **f** and MC38 **h** rechallenge mice. *P < 0.05, *** *P* < 0.001 by two-tailed Log rank test. **i** CD8 T cell depletion. Surviving mice treated with VV-iPDL1/GM for the original left flank tumor implantation were rechallenged with 5 × 10^5^ MC38 cells at right side without or with weekly i.v. injections of anti-CD8 antibody for two times. Data are presented as means ± SD (*n* = 5 mice). ****P < 0.01 by two-tailed repeated-measures two-way ANOVA. **j**–**l** Inhibition of untreated, established tumor growth. B16-F10 melanoma cells were implanted to the left and right flanks of C57B/6 mice. The mice were intratumorally injected to the left flank tumors with indicated VVs without or with i.v. injections of anti-PD-L1 antibody on days 0, 3, and 7. **j** Individual curves are depicted for each tumor. Numbers indicate complete tumor regression out of total tumors in each group. **k** Distribution of tumor volumes determined on day 30 after virus injection; *n* = 10 mice. Bars represent mean values ± SD. *P < 0.05 by two-tailed Mann–Whitney *U* test. **l** Cumulative survival curves. Data are from two independent experiments. *P < 0.05; **P < 0.01; NS, not significant by two-tailed Log rank test.

We then tested if an intratumoral injection with VV-iPDL1/GM is able to provoke a systemic antitumor response. Groups of C57BL/6 mice bearing B16-F10 tumors were treated with various VVs, and then inoculated with luciferase^+^ B16-F10 tumors on the contralateral flank. Bioluminescence imaging (Fig. 4a, b), caliper measurement (Fig. 4c), and survival curve (Fig. 4d) showed that VV-iPDL1/GM was more potent in inhibiting the growth of rechallenged homologous B16-F10 tumors, compared to VV-GM and VV-RFP. Furthermore, intratumoral injections with VV-iPDL1/GM were also more potent in inhibiting the growth of rechallenged homologous Py230 tumors and MC38 tumors, compared to the intratumoral injections with VV-GM or VV-RFP (Fig. 4e–h). In vivo CD8 T cell depletion significantly abolished the systemic antitumor activity in VV-iPDL1/GM-treated tumor-bearing mice (Fig. 4i).

We further tested if an intratumoral injection with VV-iPDL1/GM is able to provoke a systemic antitumor response against established tumor growth. B16-F10 melanoma cells were implanted to the left and right flanks of C57B/6 mice. When tumor volumes reached ~100 mm^3^, the tumors on the left flank were injected with VV-GM, VV-iPDL1/GM, or PBS without or with i.v. injections of a neutralizing anti-PD-L1 antibody (left treated tumor volumes determined on day 10 for individual tumors are shown in Supplementary Fig. 5). Intratumor injections of VV-iPDL1/GM more efficiently inhibited the growth of the untreated, distant B16-F10 tumors than intratumor injections of VV-GM did (Fig. 4j, k). Co-injections of IgG Fc and VV-GM did not substantially enhance antitumor activities of VV-GM against treated and untreated, distant tumors (Supplementary Fig. 9). The data suggested that IgG Fc domain alone unlikely contributed to the enhanced antitumor activity of VV-iPDL1/GM. We observed that systemic injections of the neutralizing anti-PD-L1 antibody alone were unable to control the growth of the weakly immunogenic B16-F10 melanoma (Fig. 4j, k), as reported in previous studies^32^. Interestingly, coadministrations with PD-L1 antibody enhanced the systemic antitumor activity of both VV-GM and VV-iPDL1/GM. However, coadministrations of PD-L1 antibody and VV-iPDL1/GM had more potent systemic antitumor activities than coadministrations of PD-L1 antibody and VV-GM (Fig. 4j–l, Supplementary Fig. 10). Collectively, these in vivo data demonstrate that intratumoral injections with the double-armed VV-iPDL1/GM alone or in combination with an anti-PD-L1 antibody are able to provoke potent, systemic antitumor responses.

### Enhanced tumor infiltration and activation of immune cells

**Fig. 5:**
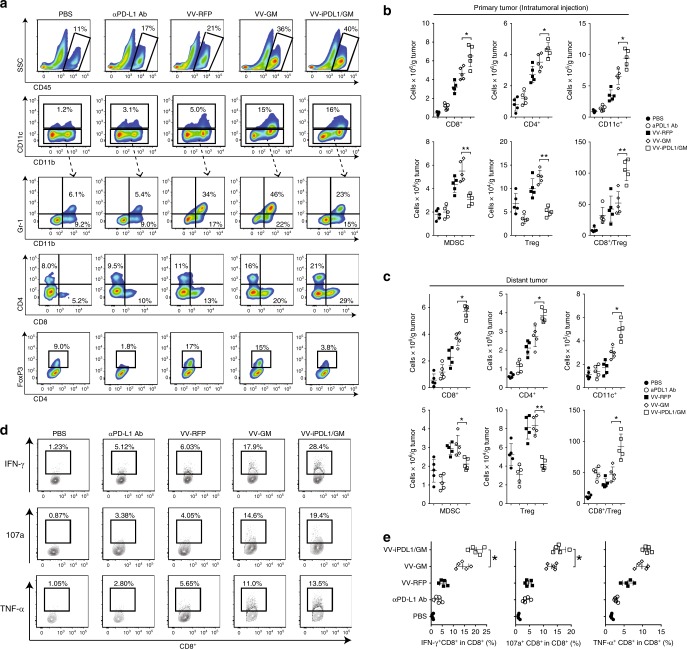
Enhanced tumor infiltration and activation of immune cells. A similar treatment schedule as in Fig. 2c was used, except that 5 days after the second VV injection, VV-treated MC38 tumors were harvested, weighed, and digested for preparation of single-cell suspensions followed by antibody staining against CD45, CD8, CD4, CD11c, CD11b, Gr-1, and FoxP3. **a**–**c** Representative plots of the percentages of infiltrating CD45^+^ immune cells, DCs, MDSCs, CD4^+^ T cells, CD8^+^ T cells, and Tregs in treated tumors **a**. Absolute numbers of the above immune cells and CD8^+^ T cell/Treg ratio values in treated tumors **b** and distant, untreated tumors **c**. *n* = 5 mice. Data presented as the means ± SD. *P < 0.05, **P < 0.01 by two-tailed Student’s *t*-test. **d**, **e** Expression of IFN-γ, TNF-α, and CD 107a of tumor-infiltrating CD8^+^ T cells in response to restimulation with MC38 tumor lysate-pulsed DCs in the presence of Golgi-plug for 8 h were measured by intracellular staining **d**. **e** Quantitative presentation of **d**. *n* = 5 mice. Data presented as the means ± SD. *P < 0.05 by two-tailed Student’s *t*-test.

We analyzed the tumor infiltration of immune cells after intratumoral injections of VV-iPDL1/GM. Groups of MC38 tumor-bearing mice were treated with various VVs via intratumoral injections. One group of MC38-bearing mice was i.p. injected with anti-PD-L1 Ab (clone 10F.9G2) for comparison. VV-intratumoral injections significantly enhanced the tumor infiltration of CD45^+^ hematopoietic cells, especially the injections of VV expressing GM-CSF (Fig. 5a). VV-GM injection enhanced composition of MDSC-containing cells (CD11b^+^Gr-1^+^, 46%) in the CD11b^+^ population. In contrast, VV-iPDL1/GM injection greatly reduced MDSCs to 23% of the CD11b^+^ population (Fig. 5a), which was consistent with the reduced absolute MDSC numbers of VV-iPDL1/GM-treated or distant tumors (Fig. 5b, c), suggesting the ability of VV-iPDL1/GM to block the PD-1/PD-L1 interaction and decrease tumor-associated immune suppressive cells. Moreover, VV-iPDL1/GM significantly enhanced dendritic cell (DC; CD11c^+^) content in the infiltrates compared with control VV-RFP (Fig. 5b, c).

We subsequently analyzed infiltrating lymphocytes in VV-treated tumors. Consistent with the published studies^23,24^, VV injections enhanced the overall lymphocyte infiltration into tumor tissues (Fig. 5a). However, the double-armed VV-iPDL1/GM enhanced the percentages of CD8^+^ T cells, and CD4^+^ T cells, and PD-1^+^CD8^+^ T cells in the CD45^+^ infiltrates more significantly in comparison to control VV or single-armed VV-GM (Fig. 5a, Supplementary Fig. 11). The injection of VV-GM alone did not significantly affect Treg cells (CD4^+^FoxP3^+^) in tumor infiltrates, but the injection of VV-iPDL1/GM reduced Treg cells to a level lower than that in PBS-treated tumors, resulting in a robustly enhanced CD8^+^ T cells/Treg ratio (Fig. 5b). We further analyzed infiltrating lymphocytes in distant, untreated tumors. Figure 5c also shows that the intratumoral injection with VV-iPDL1/GM enhanced the tumor infiltration and activation of lymphocytes and other immune cells in distant, untreated tumors. Moreover, tumor-infiltrating CD8^+^ effector T cells were more efficiently activated by VV-iPDL1/GM injections, as manifested by an enhanced expression of IFN-γ, TNF-α, and CD107a in response to the stimulation with tumor lysate-pulsed DCs (Fig. 5d, e). Altogether, these findings demonstrate that the double-armed VV-iPDL1/GM has the ability to alter the tumor microenvironment by enriching the tumor infiltration of immune cells, reducing immune suppressive cells in the tumors, and activating tumor-infiltrating effector T cells.

### Enhanced T cell responses against dominant and subdominant neoantigen epitopes

**Fig. 6:**
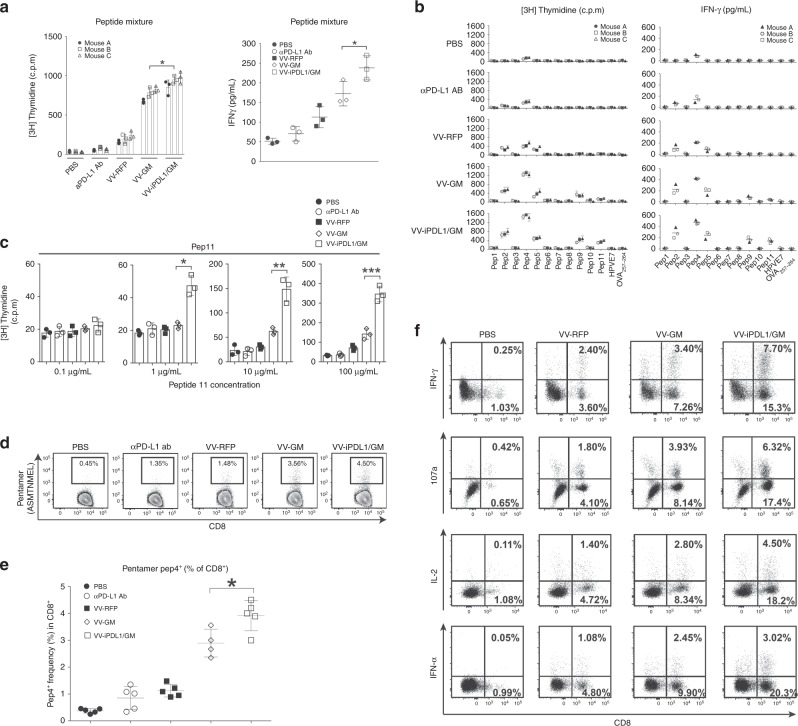
Enhanced T cell responses against dominant and subdominant tumor neoantigen epitopes. **a** Enhanced T cell responses against a pool of neoantigen peptides. MC38 tumor-bearing mice were intratumorally injected with various VVs at days 0, 3, and 7. One group of C57BL/6 mice were i.v. injected with 200 μg of anti-PD-L1 antibody. Ten days later, splenocytes were cultured in the presence of a mixture of 11 neoepitope peptides (10 μg/mL/each). After 80 h of incubation, supernatants were collected for IFN-γ ELISA (right). [^3^H] thymidine incorporation was measured (left). The graph shows the results from three mice of each group. Data presented as the means ± SD. *P < 0.05 by two-tailed Student’s *t*-test. **b** Enhanced T cell responses against individual neoantigens. The splenocytes from VV-treated mice were cocultured with each of the 11 neoepitope peptides (100 μg/ml) as above described above. [^3^H] thymidine incorporation (left) and ELISA IFN-γ concentrations (right) are shown; *n* = 3 mice. One bar or one dot represents one mouse. Data presented as the means ± SD. *P < 0.05 by two-tailed Student’s *t*-test. **c** Enhanced T cell responses against the neoantigenic peptide 11. The splenocytes isolated from VV-treated mice were cocultured with various concentrations of the neoepitope peptide 11 as above described above. [^3^H] thymidine incorporation was used to analyze T cell proliferation; *n* = 3 mice. Data presented as the means ± SD. *P < 0.05, **P < 0.01, ***P < 0.001 by two-tailed Student’s *t*-test. **d**, **e** Enhanced tumor infiltration of neopeptide 4-specific T cells. Tumor cell suspensions from various VV-treated mice using the same treatment schedule as Fig. 5a were stained with the neopeptide 4 (Pep4, ASMTNMEL)-loaded, H-2D^b^-labeled pentamers, anti-CD45, and anti-CD8. Data are representative of five independent experiments. **d** Dot plots of flow cytometry; **e** quantification of peptide 4-pentamer^+^ CD8^+^ T cells. Data presented as the means ± SD. *P < 0.05 by two-tailed Student’s *t*-test. **f** Enhanced generation of neopeptide-specific memory T cells. Forty days after the virus injection, splenocytes were restimulated with neopeptide 4-loaded DCs in the presence of Golgi-plug followed by surface staining with anti-CD8 and intracellular staining with anti-107a, anti-IFN-γ, anti-IL-2, and anti-TNF-α.

We tested whether an intratumoral injection of VV-iPDL1/GM is able to generate neoantigen-specific T cell responses. Recently, Yadav et al. identified MHC-I-restricted neoepitopes in MC38 tumor cells using whole-exome and transcriptome sequencing analysis combined with mass spectrometry^33^. MC38 tumor-bearing mice were intratumorally treated with various VVs. Ten days after the last viral injection, splenocytes were harvested and analyzed for the neoepitopes-specific immune responses. Eleven mutant neoantigen epitopes were synthesized and used for this study (Supplementary Table 1). Neopeptides 1–6 were detected on the cell surface by the membrane protein purification and mass spectrometry method, while neopeptides 7–11 were not detected on the cell surface, probably due to the sensitivity of the detection method, or poor peptide processing and presentation^33^. After intratumoral injections with VVs, the tumor-bearing mice exhibited an enhanced proliferation and cytokine (IFN-γ) secretion of splenic T cells compared with that in PBS-treated mice in response to stimulation with the 11 neopeptides mixture. However, the most potent splenic T cell responses against the neopeptides mixture were detected in the VV-iPDL1/GM-treated mice (Fig. 6a). Importantly, systematical (i.p.) administration of anti-PD-L1 antibody (200 μg) did not significantly induce neopeptide-specific T cell responses in the tumor-bearing mice. These data indicate the superior potency of VV-iPDL1/GM to activate neoantigen-specific T cell responses.

We then analyzed T cell responses in VV-treated mice against individual neoepitopes. VV-RFP enhanced the proliferation and cytokine production of splenic T cells of treated mice in response to neoepitopes 2, 4, and 5, compared to only neoepitope 2 or 4 in the PBS or anti-PD-L1 antibody-treated mice (Fig. 6b, Supplementary Fig. 12). VV-GM significantly enhanced the T cell responses to neoepitopes 2, 4, and 5, and also additionally triggered T cell responding to neoepitope 9 and slightly to neoepitope 11. Compared with VV-GM, VV-iPDL1/GM further strengthened T cell responses against neoepitopes 2, 4, and 5, as well as the subdominant neoepitopes 9 and 11 (Fig. 6b, Supplementary Fig. 12). Furthermore, splenic T cells from the VV-iPDL1/GM-treated mice showed responses against dominant neoepitope 2, 4, or 5 even at a very low peptide concentration (0.1 μg/mL or 1 μg/mL), and also showed responses against subdominant neoepitope 9 or 11 at a low concentration (10 μg/mL), in which splenic T cells from other VV-treated mice didn’t show detectable responses (Fig. 6c, Supplementary Fig. 12). Given the prominent neoepitope 4-specific T cell response detected in various VV-treated mice, neoepitope 4 peptide-MHC H-2D^b^-labeled pentamers were synthesized and used to analyze tumor-infiltrating neoantigen-specific T cells. Among the groups, the VV-iPDL1/GM-treated mice had maximal CD45^+^CD8^+^pentamer^+^ T cells in tumor infiltrates (Fig. 6d, e), indicative of VV-iPDL1/GM injections being most efficacious in activating neoepitope 4-specific T cells in the tumor-bearing mice. Even 40 days after the last VV injection when all tumors were gone, splenocytes of the VV-iPDL1/GM-treated mice showed the strongest response to neoepitope 4-loaded DC restimulation (Fig. 6f). These results demonstrate the ability of VV-iPDL1/GM to activate T cell responses against dominant and subdominant neoantigen epitopes.

### Enhanced tumor-infiltrating DC maturation and neoantigen presentation

**Fig. 7:**
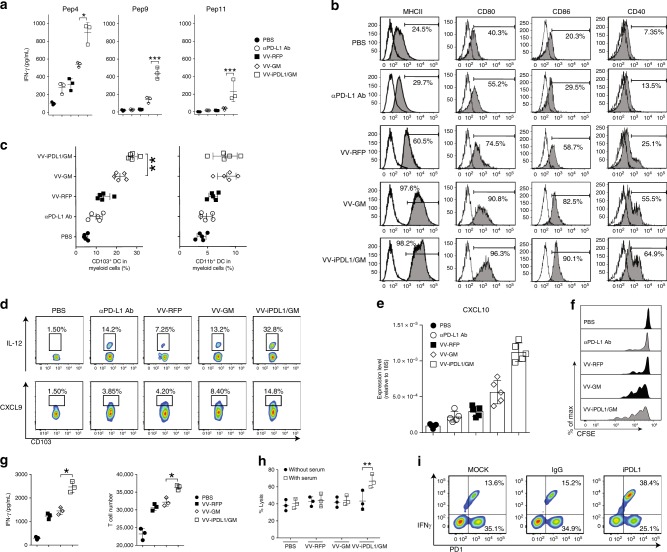
Enhanced neoantigen presentation and cytolytic activity of neoantigen-specific CTLs. **a** Enhanced stimulatory potency of tumor-infiltrating DCs. Tumor-infiltrating DCs from VV-treated mice were loaded with neopeptide 4, 9, or 11, and cocultured with the neoantigens-primed T cells from mice immunized with the 11 neopeptide mixture to assess IFN-γ production; *n* = 3 mice. Data presented as the means ± SD. *P < 0.05, ***P < 0.001 by two-tailed Student’s *t*-test. **b** Enhanced maturation of tumor-infiltrating DCs. Using a similar treatment schedule as described in Fig. 5a, cell suspensions prepared from VV-treated tumors were analyzed by flow cytometry. **c** Enhanced tumor infiltration of CD103^+^ DCs. Using the same treatment schedule as in Fig. 5a, tumor cell suspensions from VV-treated mice were analyzed by FACS; *n* = 5 mice. Data presented as the means ± SD. **P < 0.01 by two-tailed Student’s *t*-test. **d** Intracellular staining of IL-12 and CXCL9 of CD103^+^ DCs from VV-treated tumors. **e** qRT-PCR analysis of CXCL10 mRNA levels in CD103^+^ DCs isolated from VV-treated tumors; *n* = 5 mice. Data presented as the means ± SD. **P < 0.01 by two-tailed Student’s *t*-test. **f** Neoantigens-primed T cells proliferated more efficiently in VV-iPDL1/GM-treated mice. The neoantigens-primed T cells were labeled with 5 μM CFSE and i.v. injected into VV-treated mice. Three days later, T cell proliferation was assessed by FACS. **g** Enhanced stimulatory effect of VV-iPDL1/GM-infected tumor cells. MC38 tumor cells infected with VVs at MOI = 1 were cocultured with the neoantigens-primed T cells. IFN-γ production (left) and T cell proliferation (right) were measured. Data presented as the means ± SD. *P < 0.05 by two-tailed Student’s *t*-test. **h** Serum of VV-iPDL1/GM-treated mice enhanced the cytolytic activity of neoantigens-primed T cells. MC38-Luc cells were cocultured with the neoantigen-specific T cells in the presence of the sera from treated MC38-bearing mice. Cytolytic activity was calculated using luciferase emission value. Data are presented as means ± SD. **P < 0.01 by two-tailed Student’s *t*-test. **i** PD-1^+^ CD8^+^ T cells isolated from VV-treated MC38 tumors were cocultured with MC38 cells in the presence of purified iPDL1 or IgG. IFN-γ^+^ frequencies of PD-1^+^ T cells were shown from one of two independent experiments.

We further explored the mechanisms of the double-armed VV-iPDL1/GM to activate neoantigen-specific T cell responses. DCs are professional antigen-presenting cells with the ability to prime antigen-specific T cell responses. Thus, we compared the immunostimulatory potency of tumor-infiltrating DCs from various VV-treated mice. Tumor-infiltrating CD11c^+^ DCs isolated from VV-treated MC38 tumors were pulsed with neopeptides 4 (dominant), 9, and 11 (subdominant), and then cocultured with neoantigens-primed T cells isolated from mice immunized with the 11 neoepitope peptides mixture formulated with adjuvants. Tumor-infiltrating DCs from VV-iPDL1/GM-treated MC38 tumors had the enhanced potency to stimulate neoantigens-primed T cells (Fig. 7a). In comparison, tumor-infiltrating DCs from MC38 tumor-bearing mice receiving anti-PD-L1 antibody (i.v.) alone only had a much weaker stimulatory potency. We also observed that VV-iPDL1/GM significantly promoted tumor-infiltrating DC maturation, as evidenced by an increased expression of MHCII, CD80, CD86, and CD40 (Fig. 7b). A recent study revealed that CD103^+^ DCs are the main intratumoral myeloid cell population that transports antigens to the tumor-draining lymph nodes for activating T cells^34^. The analysis of surface markers on the DC population showed that VV-iPDL1/GM injection significantly increased tumor-infiltrating CD103^+^ DCs, compared to VV-GM or VV-RFP (Fig. 7c, Supplementary Fig. 13). IL-12 is known to be an important cytokine in cross talk between DCs and T cells^35^. Chemokines CXCL9 and CXCL10 direct effector T cell trafficking and tumor infiltration^36^. The expression of IL-12, CXCL9, and CXCL10 in CD103^+^ DCs from VV-iPDL1/GM-treated tumors was elevated (Fig. 7d, e). These data demonstrate that VV-iPDL1/GM injections likely enhanced tumor-infiltrating DC maturation and neoantigen presentation.

### Enhanced neoantigen presentation on tumor cells, and CTL effector function

During the effector phase of the antitumor response, activated T cells need to recognize neoantigen-presented tumor cells for their effector function. A poor neoantigen presentation and the expression of PD-L1 can render tumor cells resistant to CTL-mediated cytolysis. We performed an in vivo T cell proliferation assay, in which neoepitopes-primed T cells were adoptively transferred into the various VV-treated MC38-bearing mice. A higher efficiency in neoepitopes-primed T cell proliferation in vivo was observed in VV-iPDL1/GM-treated MC38-bearing mice (Fig. 7f). We tested the ability of neoantigen-specific T cells to recognize MC38 tumor cells infected with various VVs in vitro. MC38 tumor cells were infected with VV-iPDL1/GM or control VVs, and after washing, then cocultured with neoantigens-primed T cells isolated from mice immunized with the 11 neoepitope peptides mixture formulated with adjuvants. VV-iPDL1/GM-infected MC38 tumor cells were more potent in stimulating the proliferation and cytokine production of the neoepitopes-primed T cells (Fig. 7g), suggesting that the neoepitopes-primed T cells more efficiently recognize and interact with the neoepitopes-presented, VV-iPDL1/GM-infected tumor cells. We further tested the role of the secreted iPDL1 in enhancing tumor cell immunogenicity. MC38 tumor cells without VV infection were cocultured with the neoepitopes-primed T cells isolated from mice immunized with the 11 neoepitope peptides mixture in the presence of sera from tumor-bearing mice treated with various VVs. Figure 7h shows that only sera from VV-iPDL1/GM-treated mice were able to enhance the cytolytic activity of neoantigens-primed T cells against various VV-infected MC38 tumor cells. Moreover, it was observed that higher IFN-γ^+^ frequency of PD-1^+^ CD8^+^ T cells isolated from VV-treated MC38 tumor cell suspensions in the in vitro coculture with MC38 tumor cells in the presence of purified iPDL1 in comparison to the presence of control IgG (Fig. 7i), suggesting the role of secreted iPDL1 in overcoming the immunosuppression of PD-L1^+^ tumor cells. In addition, our preliminary data showed the upregulation of the expression of TNF signaling genes and protein processing genes in VV-iPDL1/GM-infected tumor cells by RNA-Seq and qRT-PCR (Supplementary Fig. 14, Supplementary Table 2). A possible role of neoantigen presentation enhanced by VV-iPDL1/GM infection still cannot be ruled out. These data demonstrate the enhanced neoantigen presentation on tumor cells by VV-iPDL1/GM infection for enabling neoantigen-specific CTL effector functions.

## Discussion

T cells against mutant neoantigens that are individually tumor specific play a critical role in driving antitumor immunity. Each tumor harbors a unique repertoire of mutated neoantigenic peptides that are immunogenic and can potentially induce tumor-specific immune responses. T cells can be activated against shared, nonmutated tumor-associated self-antigens. NK cells and NKT cells also have antitumor activities. Thus far, the majority of cancer patients still fail to spontaneously activate neoantigen-specific T cells and are resistant to immune checkpoint blockade therapy, likely due to the poor presentation of tumor neoantigens and the immunosuppressive tumor microenvironment. Thus, the activation of tumor neoantigen-specific T cells is critical to enhancing the efficacy of tumor immunotherapy.

The results of this study demonstrate that the armed oncolytic virus coexpressing a PD-L1 inhibitor and GM-CSF (VV-iPDL1/GM) was able to produce the PD-L1 inhibitor and systematically bind to PD-L1^+^ tumor cells and immune cells. The intratumoral injections with VV-iPDL1/GM produced iPDL1, enhanced neoantigen presentation, and activated systemic T cell responses against dominant, as well as subdominant neoantigens, resulting in the effective rejection of both virus-injected and distant tumors. Thus, this double-armed oncolytic virus is capable of activating neoantigen-specific T cell responses by the likely synergistic action of PD-L1 inhibition on tumor cells and immune cells, viral replication, and GM-CSF stimulation.

Individual tumors with numerous genetic mutations can contain high numbers of potentially immunogenic neoantigens^37–39^. Despite the presence of immunogenic neoantigen epitopes in each tumor, spontaneous priming and activation of neoantigen-specific T cells are inefficient in the majority of cancer patients. Immunosuppressive tumor microenvironments, due to the lack of the “danger signals” of pathogen-associated molecular pattern (PAMP) molecules, and the expression of immune checkpoints, such as PD-L1, on tumor cells, T cells, and DCs, inhibit the priming or activation of T cell responses against tumor neoantigens^40–42^. The engineered oncolytic virus generated in this study is able to produce the PD-L1 inhibitor, and bind to PD-L1^+^ tumor cells and immune cells. It is tempting to postulate that the secretory iPDL1 in combination with the viral oncolysis-mediated, immunogenic cell death and the release of viral PAMP molecules from infected cells may lead to the enhanced DC maturation and neoantigen presentation in the tumor microenvironment, and the systemic activation of tumor neoantigen-specific T cell responses. Thus, this study demonstrates that secretory PD-L1 inhibitors, GM-CSF, and viral oncolysis work synergistically to promote neoantigen presentation and activate tumor neoantigen-specific T cell response, representing a potent, individual tumor-specific oncolytic immunotherapy.

An interesting finding of this study is the ability of the armed oncolytic virus to activate T cell responses against subdominant neoantigen epitopes. T cell responses are primed or activated by DCs, which present a repertoire of MHC-associated peptides. The tumor neoantigen repertoire derived from mutated gene products are presented to T cells after DCs capture and process antigens, load processed peptides onto MHC-I molecules via cross-presentation, and go through the maturation processing associated with the upregulation of costimulatory molecule and cytokine expression triggered by PAMP molecules^43–46^. It is postulated that mutated proteins are processed and presented by the MHC molecule as neoantigens to T cells at different levels of efficacy such that certain mutated epitopes are efficiently processed and presented (dominant neoantigen epitopes), whereas others are poorly processed and presented at subthreshold levels, especially the microenvironment with PD-L1 expression on APCs (subdominant or cryptic neoantigen epitopes). The synergistic action of viral oncolysis, GM-CSF, and PD-L1 inhibition of DCs and T cells by this engineered oncolytic virus may enhance the ability of DCs to present the neoantigen repertoire to T cells, leading to the activation of T cell responses against both dominant and subdominant neoantigenic epitopes.

During the effector phase of antitumor T cell responses, the poor processing and presentation of neoantigenic epitopes and the expression of PD-L1 on tumor cells can inhibit CTL effector functions. The results of this study demonstrated that an intratumoral injection with this engineered oncolytic virus promoted the tumor infiltration and activation of neoantigen-specific T cells and immune cells, as well as neoantigen presentation on tumor cells via the inhibition of PD-L1 by the secreted PD-L1 inhibitors, leading to the systemic rejection of both the treated tumor and distant tumors.

In earlier studies from our groups and others, oncolytic viruses were armed with immune stimuli, such as heat shock proteins, chemokines, and cytokines, to activate antitumor immune responses^19,20,47–52^. However, oncolytic virus therapies so far only showed limited efficacy in cancer patients. Several recent studies reported the generation of armed oncolytic viruses expressing PD-1/PD-L1 inhibitors for activating antitumor immune responses. An armed oncolytic adenovirus expressing an anti-human PD-L1 mini-body was found to enhance CAR T cell killing activities in human tumor xenograft models^53^. An oncolytic HSV expressing a PD-1 scFv was found to induce a durable antitumor response in preclinical mouse models of GBM (ref. ^54^). A recombinant myxoma virus (vPD1) that expresses a PD-1 extracellular domain was found to inhibit the PD-1/PD-L1 pathway and activate antitumor immunity^55^. However, these studies did not investigate the ability and mechanisms of the armed oncolytic viruses to activate tumor neoantigen-specific T cell responses. Recent studies found that the reactive upregulation of PD-L1 expression in the tumor microenvironment after virus administration caused the tumor resistance to oncolytic immunotherapy^23,24^. The production of PD-L1 inhibitors by this engineered oncolytic virus generated in this study should overcome this problem. Moreover, this oncolytic virus, which activates the neoantigen-specific T cell response by the synergistic action of PD-L1 inhibition, GM-CSF, and viral oncolysis in the tumor microenvironment may be advantageous to the therapies with PD-1/PD-L1 antibodies.

In summary, this engineered armed oncolytic virus with the ability to activate neoantigen-specific T cell responses by the synergistic action of viral immunogenic oncolysis, GM-CSF function, and PD-L1 inhibition on tumor cells and immune cells provides a potent, individual tumor-specific oncolytic immunotherapy, which could be therapeutically used alone or in combination with immune checkpoint inhibitors, targeted therapy, and chemotherapy for cancer patients, especially those resistant to PD-1/PD-L1 blockade therapy.

## Methods

### Cell lines

Human embryonic kidney cell line 293T, osteosarcoma HUTK-143B, monkey kidney fibroblasts CV1, murine adenocarcinoma Py230, murine melanoma B16-F10, and murine lymphoma EL4 were purchased from the American Type Culture Collection (ATCC). Murine colon adenocarcinoma cells MC38 was purchased from Kerafast. All the adherent cells were cultured in complete Dulbecco’s modified Eagle’s medium supplemented with 10% heat-inactivated fetal bovine serum (FBS) and 1% penicillin–streptomycin–glutamine 100× (Thermo, cat. no.: 10378016). T cells and splenocytes were grown in RPMI with 10% of heat-inactivated FBS, 10 mM HEPES, 1 mM sodium pyruvate, 0.05 mM β-mercaptoethanol, 1% penicillin–streptomycin–glutamine, and 1× minimal essential medium nonessential amino acids. Cells were maintained in an incubator at 37 °C and 5% CO_2_.

### Antibodies

The antibodies used in the study included: anti-CD16/32 (clone: 93, Biolegend, 1:100), anti-PD-L1 (APC or PE-cy7, clone: 10F.9G2, Biolegend; clone: MHI5, eBioscience, 1:100), anti-IgG2a-Fc (Polyclonal, Thermo, 1:500), anti-CD45 (BV421 or PE, clone: 30-F11, Biolegend, 1:500), anti-CD11c (PE or APC, clone: HL3, BD Biosciences, 1:100), anti-CD11b (eF450 or PE-cy5, clone: M1/70, BD Biosciences, 1:100), anti-CD103 (FITC, clone: 2E7, Biolegend, 1:100), viability dye (BV510 or UV450, Tonbo Biosciences, 1:1000), anti-CD3 (FITC or Pacific Blue, clone: 17A2, Biolegend, 1:1000), anti-CD4 (PE or PE-cy5, clone: RM4-5, BD Biosciences, 1:500), anti-CD8 (FITC, APC, or APC-cy7, clone: 53-6.7, Biolegend, 1:1000), anti-Gr-1 (PE or PE-cy7, clone: RB6-8C5, Biolegend, 1:100), MHCII (Pacific Blue, FITC, or PE, clone: M5/114.15.2, Biolegend, 1:100), anti-FoxP3 (APC or PE, clone: FJK-16s, Biolegend, 1:100), anti-IFN-γ (APC, clone: XMG1.2, Biolegend, 1:100), anti-107a (FITC, clone: 1D4B, Biolegend, 1:500), anti-TNFα (PE, clone: MP6-XT22, BD Biosciences, 1:100), anti-IL-2 (PerCP^−^cy5.5, clone: JES6-5H4, Biolegend, 1:100), anti-IL-12 (PE-cy7, clone: C15.6, Biolegend, 1:100), anti-CXCL9 (AF647, clone:MIG-2F5.5, Biolegend, 1:100), anti-CD80 (PE-cy5, clone: 16-10A1, Biolegend, 1:100), anti-CD86 (PE-cy7, clone: GL-1, Biolegend, 1:100), and anti-CD40 (PE, clone 3/23, Biolegend, 1:100), anti-CD31 (FITC, clone: 390, Biolegend, 1:100), anti-Ter119 (APC, clone: TER-119, Biolegend, 1:100), IRDye® 800CW Goat anti-Mouse IgG Secondary Antibody (Polyclonal, Li-cor), anti-CD40 (cat. no.: BP0016-2, Lot: 671717N1, BioXcell), and anti-PD-L1 (clone: 10F.9G2, BioXcell) Pentamer H-2Db-ASMTNMELM-PE was provided by ProImmune Inc (1:100).

### Recombinant VV generation and purification

VV shuttle vector pSel-DsRed2N1 (refs. ^25,26^) was used to construct the recombinant shuttle vectors for coexpressing iPDL1 (Supplementary Fig. 15a, b) under the control of the VV Pse/l promoter and GM-CSF under the control of the VV p7.5 later early promoter (Fig. 1a). To generate recombinant ddVVs, a vgf gene-deleted WR strain VV, vSC20 was used as a parental virus for homologous recombination. In brief, CV1 cells were infected with vSC20 at multiplicity of infection (MOI) of 0.1 for 2 h and then transfected with one of the recombinant shuttle plasmids. Cell extraction solution was used to infect HUTK-143B cells in the presence of 50 μg/mL bromodeoxyuridine (Sigma B5002). Three RFP-positive plaques were isolated, resuspended and further infect HUTK-143B cells for three more cycles of plaque selection until all plaques were RFP positive^25,56^. The dislodged virus-infected cells were harvested with supernatants discarded by 5 min 1000 × *g* centrifugation. The cells resuspended in 1–2 mL chilled 10 mM Tris buffer (pH = 9.0) were sonicated for 1 min in water bath, and frozen/thawed for three times in dry ice/ethanol bath. The nucleus-free cell lysate was carefully layered in an ultracentrifuge tube appropriate for an ultracentrifuge SW41 rotor prelayered with 2 mL of a 40% sucrose solution, and centrifuged at 20,000 × *g* for 2 h at 4 °C without brakes. The white pellets at the bottom of the tube after ultracentrifugation resuspended in 200 μL to 1 mL 10 mM Tris buffer were stored at −80 °C and further used for animal study^56^.

Titration of viruses: HuTK-143B cells (2 × 10^5^) were seeded into 12 well plates for 24 h. VVs with tenfold serial dilutions were added onto the cell monolayer. After 1 h incubation with rocking, the cells were gently added with 2 mL culture media and incubated for 24–48 h. The cells were washed and fixed in 0.1% crystal violet in 20% ethanol. The plaques were counted under microscope^57^.

### Western blot

A total of 5 × 10^6^ cells cultured in six-well plates were infected with indicated VVs at MOI = 2. After incubation for 48 h, supernatants were harvested and clarified by centrifugation at 10,000 × *g* for 2 min. Cells were lysed in 1× RIPA buffer (Sigma-Aldrich, St Louis, MO) and 1× mammalian protease inhibitor (Sigma-Aldrich, St Louis, MO) for 15 min on ice and clarified by centrifugation at 10,000 × *g* for 2 min. Samples of both supernatants and cell lysates were mixed with 6× sodium dodecyl sulfate (SDS) sample buffer (Bioworld, Dublin, OH) and electrophoresed in a 4–20% gradient SDS–polyacrylamide gel (Thermo, Waltham, MA). The fractionated protein samples are transferred onto 0.2 μm nitrocellulose membrane (Thermo, Waltham, MA). The nitrocellulose membrane is blocked for 30–60 min at room temperature (RT) in TBS buffer (Bio-Rad, Irvine, CA) containing 5% nonfat milk. Immunodetection of iPDL1 is performed by incubation with RD800-conjugated goat anti-mouse IgG antibody (Licor, Lincoln, NE) at RT for 1 h, or with rat anti-mouse PD-1 (Biolegend, San Diego, CA) at 1 µg/mL for overnight at 4 °C followed by with an RD800-conjugated anti-Rat IgG (Licor, Lincoln, NE). The blots are detected with an Odyssey Imager (LI-CON, Lincoln, NE).

### Enzyme-linked immunosorbent assay

Tumor cells were infected with indicated viruses at MOI = 2. After 24, 48, or 72 h, supernatants of the tumor cell cultures were collected. Mouse serum was collected at indicated times after intratumoral injection of indicated VVs. Serum iPDL1 or GM-CSF was determined using mouse PD-1 DuoSet ELISA kit (R&D, Minneapolis, MN) or mouse GM-CSF ELISA kit (Biolegend, San Diego, CA).

### MTT assay

Tumor cells seeded in a 96-well plate were infected with indicated VVs at various MOIs in triplicate. The viability of tumor cells was determined using MTT assay (ATCC, Manassas, VA) following the manufacturer’s instruction. Optical density was read at 490 nm wavelength on a VersaMax microplate reader. The viability of the infected tumor cells was calculated as a percentage relative to the mock-infected cells^58^. Data = mean ± SD.

### BM-derived DC differentiation assay

Freshly isolated BM cells from mice were cultured in complete RPMI1640 media supplemented with 10% FBS, 20 ng/mL GM-CSF, and 40 ng/mL IL-4 for 72 h. Adherent or loosely adherent cells were collected, resuspended in culture media supplemented with 100 ng/mL IL-4 (Peprotech, London, UK), and aliquoted into 12-well tissue culture plate. A total of 100 μL of the supernatants of various VVs-infected cells (0.1 μm filtered) were added. A total of 50 ng/mL commercial GM-CSF (Peprotech, London, UK) was added as a positive control. All the cells were cultured for an additional 72 h and then analyzed by flow cytometric staining with APC-anti-CD11c (ref. ^59^).

### iPDL1 protein purification

HUTK-143B cells were infected with VV-iPDL1/GM at MOI = 2 without FBS. Culture media was collected 48 h post infection, and filtered by 0.8 μm syringe filter unit (Millipore, Darmstadt, Germany). The media was incubated with 200 μL Protein G Sepharose (Sigma-Aldrich, St Louis, MO) at 4 °C overnight. The protein G beads were washed by 1× PBS three times, and eluted by 0.1 M glycine-HCL, pH=2.8. The elution was dialyzed in 4 L 1 × PBS overnight^30,31,60^. The concentration of the iPDL1 protein was determined using BSA Assay kit (Thermo, Waltham, MA).

### iPDL1 binding assay by flow cytometry

Tumor cells were infected with PBS, VV-RFP, and VV-iPDL1/GM at MOI = 0.5. 24 h later, all cells were collected, and stained with anti-IgG Fc. For some experiments, tumor cells were first cultured for overnight in the presence of IFN-γ (20 ng/mL) to enhance PD-L1 expression and then infected with PBS, VV-RFP, and VV-iPDL1/GM at MOI = 0.5. Forty hours later, all the cells were collected, and stained with anti-PD-L1 or anti-IgG Fc. PD-L1^+^RFP^+^ (virus-infected) cells and PD-L1^+^RFP^−^ (uninfected) cells were analyzed by flow cytometry. For detecting purified iPDL1 binding, wild-type MC38 cells and MC38 cells transduced with the recombinant lentiviral vector PD-L1shRNA/GFP were incubated with 50 μg/mL IgG or purified iPDL1 for 30 min on ice. Cells were then stained with anti-PD-L1 or anti-IgG Fc.

### Inhibition of PD-1/PD-L1 interaction

Ninety six-well ELISA plates were coated with 1 μg/well PD-L1 protein (Abcam, ab130039). A total of 50 μL mixture of 20 ng mouse PD-1-biotin (Sino Biological, 50124-M08H-B) and purified iPDL1, IgG control (Sigma, I5381), or anti-PD-L1 antibody control (Biolegend, 124301) at indicated concentration, or 50 μL assay buffer (blank) was added into wells, and incubated at RT for 2 h. Diluted streptavidin–HRP was added to each well after wash and incubated at RT for 1 h with slow shaking. After three times of wash, TMB HRP substrate was added until blue color is developed in the positive control well. OD value at 450 nm UV was measured after 100 μL 2N sulfuric acid was added to stop reaction. (OD of unknown − OD of blank)/(OD of positive control − OD of blank) represents the percent of inhibition activity.

### ADCC assay

A total of 1 × 10^4^/well target cells MC38 or IFNγ-stimulated MC38 cells were seeded in a 96-well plate 1 day before the experiment. On the day of experiment, different amounts of PBS, IgG Fc (Thermo, cat. no.: 31205) or purified iPDL1 were added into wells containing target MC38 cells followed by the addition of 6 × 10^4^ ADCC bioassay effector cells per well that were provided in the ADCC Reporter Bioassays kit (Promega, Madison, WI). After incubation for 6 h at 37 °C, the plates were kept on the bench for 15 min. Then each well was added with 75 μL of Bio-Glo Luciferase reagent and kept at RT for 10 min. Luminescence values were measured using a plate reader with glow-type luminescence read capabilities.

### Mouse experiments

All the animal experiments were performed in accordance with the guidelines of the Institutional Animal Care and Use Committee of USC, and were bred and maintained in our institute-specific pathogen-free facilities. B16-F10, B16-F10-Luc, Py230, or MC38 tumors were established by subcutaneously injecting 5 × 10^5^ of corresponding tumor cells into the left flank of C57BL/6J mice (*N* = 5 or 10 per group, the Jackson Laboratory). For the established tumor model, 1 × 10^5^ B16-F10 cells were injected to the right flank simultaneously. When left flank tumor sizes reached ~100 mm^3^ or indicated sizes, tumors were intratumorally injected with 50 μL of the indicated VVs three times on days 0, 3, and 7 (5 × 10^7^ pfu/tumor), or PBS with or without i.v. injections of 50 μL (200 μg/mL) of anti-PD-L1 antibody. Tumor sizes of treated primary tumors and untreated tumor on the contralateral side (distant tumor) were measured by caliper or monitored by bioluminescence imaging for B16-F10-Luc tumors. Tumor volumes were calculated according to the formula: width^2^ × length × 0.5. For tumor rechallenge assay, the treated mice were subcutaneously injected with 2.5 × 10^5^ B16-F10 cells, 5 × 10^5^ Py230, or 5 × 10^5^ MC38 onto the right flank of each mouse at indicated days after the virus treatment. A group of naive mice were injected with tumor cells for control. The rechallenged tumors were monitored as above described. For CD8 T cell depletion experiment, anti-CD8 antibodies (clone: 2.43, Bio X cell, cat. no.: BP0061) were injected i.v. twice weekly starting one day prior to viral injection.

### Neoantigen-specific T cell response assays

Splenocytes were isolated from various VV-treated tumor-bearing C57BL/6 mice and cultured in a 96 round bottom well plate (1 × 10^5^ cells/well) in the presence of single neopeptide or a mixture of neopeptides of MC38 at the indicated concentrations at 37 °C in 5% CO_2_. After 80 h incubation, 200 μL supernatants were collected from each well to evaluate IFN-γ via ELISA. [^3^H] thymidine (1 μCi per well) was added and cultured for an additional 16 h. [^3^H] thymidine incorporation was measured in TopCount Scintillation and Luminescence Counter. For flow cytometric analysis, splenocytes from the various VV-treated groups were cocultured with syngeneic monocyte-derived DCs (10:1) that were pulsed with neopeptides for 12 h in the presence or absence of Golgi-plug^61^. Cells were stained with anti-CD8, anti-107a, anti-IFN-γ, anti-IL2, and anti-TNF-α, and analyzed by flow cytometry.

### Virus replication assay in vivo

C57BL/6 mice were implanted with 5 × 10^5^ MC38 cells subcutaneously. When the tumors reached ~100 mm^3^, mice were treated with 1 × 10^7^ pfu/mouse VV-iPDL1/GM intratumorally. On indicated days, mice were killed and tissues were subjected to three cycles of freeze-thaw-sonication to release virus. A total of 500 μL homogenate were incubated on 143B TK cells and titers were determined^25,26^. Viral titers were standardized to tissue weight.

### Generation of neoepitopes-primed T cells

C57BL/6 mice (6–8 weeks) were injected intraperitoneally with a mixture of 11 peptides (10 μg each) formulated with the adjuvant system consisting of 100 μg anti-CD40 (Abclone FJK45) and 100 μg poly (I:C; InvivoGen) two times on days 0 and 14. On day 21, the splenocytes were harvested and in vitro stimulated with irradiated autologous naive splenocytes prepulsed with the peptide mixture for two rounds. Expanded splenic cells were harvested for further experiments^33,62^.

### Isolation of tumor-infiltrating immune cells

C57BL/6 mice were subcutaneously inoculated with MC38 cells (1 × 10^6^) on one side flank. When the tumor sizes reached ~100 mm^3^ or indicated sizes (counted as day 0), mice were intratumorally injected with 50 μL of PBS, VV-RFP, VV-GM, or VV-iPDL1/GM (5 × 10^7^ pfu/tumor) on days 0 and 3. One group of mice were intraperitoneally injected with 200 μg of anti-PD-L1 antibody (clone 10F.9G2). At indicated days post viral treatment, tumors were collected, weighed, and digested with collagenase type I and DNase for 30 min at 37 °C. The tumor tissues were homogenized and then filtered through a 70-μm nylon strainer^33^. Single-cell suspensions were analyzed by FACS or used for other assays^61^.

### In vitro and in vivo assays of neoepitopes-specific T cell responses

Tumor-infiltrating DCs from various VV-treated, tumor-bearing mice were isolated using CD11c MicroBeads UltraPure (Miltenyi Biotec, 130-108-338). The DCs were pulsed with indicated neopeptides, and then cocultured with the neoantigens-primed T cells to assess cytokine production and T cell proliferation^61^.

To assess the immunogenicity of the VV-treated tumor cells, MC38 cells seeded in 96-well round bottom plates (5 × 10^3^ per well) were infected with PBS, VV-RFP, VV-GM, or VV-iPDL1/GM at MOI = 1 for 2 h. Infected MC38 cells were extensively washed and then cocultured with 2 × 10^4^ the neoantigens-primed T cells for 48 h. One of mock-infected MC38/CTL cocultures was added with 1 μg/mL anti-PD-L1 antibody. Supernatants were harvested for analyzing IFN-γ production via ELISA. Cells were harvested, immune stained with anti-CD3. T cell numbers were counted by adding precision counting beads (Biolegend, 424902).

To assess in vivo proliferation of the neoantigens-primed T cells in various VV-treated, tumor-bearing mice, MC38 tumor-bearing mice were treated with the indicated viruses (5 × 10^7^ pfu), followed by adoptive transfer of CFSE-labeled 2 × 10^6^ the neoantigens-primed T cells. Three days later, TdLNs were harvested and their proliferation was assessed based on CFSE dilution via flow cytometry^34^. Data shown are a representative histogram of two independent experiments.

### CTL assay

Firefly Luciferase stably expressing cells were cocultured with effector T cells at the indicated ratios. Forty eight hours later, all the cells were spun down and resuspended in 100 μL media supplemented with 100 μg/mL Beetle Luciferin Potassium Salt and incubated at RT for 5 min. Cells were transferred to 96-well white opaque plate. Luciferase emission was measured on a TopCount Scintillation and Luminescence Counter. Killing lysis % = [1 − (unknown − blank)/(positive control − blank)] × 100%.

### RNA sequencing

MC38 cells were infected with VV-iPDL1/GM. Cells were harvested at various times. Cellular RNAs were extracted from cell lysates using RNeasy Plus Mini Kit (Qiagen). Total RNA is enriched by oligo (dT) magnetic beads (rRNA removed). RNA-seq library preparation using KAPA Stranded RNA-Seq Library Prep Kit (Illumina). The libraries were sequenced on a HiSeq 4000 instrument using 2 × 150 bp pair-end sequencing (Arraystar Inc, Rockville, MD).

### Software

Odyssey v3.0, MikroWin2000, Living Image v4.4, FACS DIVA 6.1.2, Illustrator CS6, flowjo 10.4.0, Graphpad prism 6, Microsoft excel 2011 for mac, Living Image v4.3.1, RNA-seq analysis was performed with the following software HTSeq v0.5.3, Solexa pipeline v1.8, FastQC software 0.11.7, Hisat2 software, StringTie 1.3.3, R 3.4.1, and Python 2.7.

### Statistics

Statistical analysis was performed using GraphPad Prism 6. When passing the normality test, two-tailed Student’s *t*-test was used to compare the two groups. Otherwise, a Mann–Whitney *U* test was used. Repeated-measures two-way ANOVA with Bonferroni’s correction was used to compare the effect of multiple levels of two factors with multiple observations at each level (for tumor volumes). Animal survival is presented using Kaplan–Meier survival curves and was statistically analyzed using log rank test. The data presented in the figures are mean ± SD. *P* values < 0.05 were considered to statistically significant.

### Reporting summary

Further information on research design is available in the Nature Research Reporting Summary linked to this article.

## Supplementary information

Supplementary Information

Reporting summary

## Data Availability

The RNA-seq data have been deposited in the NCBI GEO database under the accession code GSE145823. The source data underlying Supplementary Fig. 14b are provided as a Source Data file. All the other data supporting the findings of this study are available within the article and its Supplementary Information files, and from the corresponding author upon reasonable request. A reporting summary for this article is available as a Supplementary Information file.

## Source Data

Source Data
