# Supplementary material for: An engineered oncolytic virus expressing PD-L1 inhibitors activates tumor neoantigen-specific T cell responses
Source: Nat Commun. 2020 Mar 13;11:1395. doi: 10.1038/s41467-020-15229-5 (PMC7070065; doi:10.1038/s41467-020-15229-5)
Supplement: Supplementary file 1 — Supplementary Information [file 41467_2020_15229_MOESM1_ESM.pdf]

Supplementary Information

An Engineered Oncolytic Virus Expressing PD-L1 Inhibitors Activates Tumor Neoantigen-Specific T Cell Responses

Guan Wang et al.

Supplementary Figure. 1

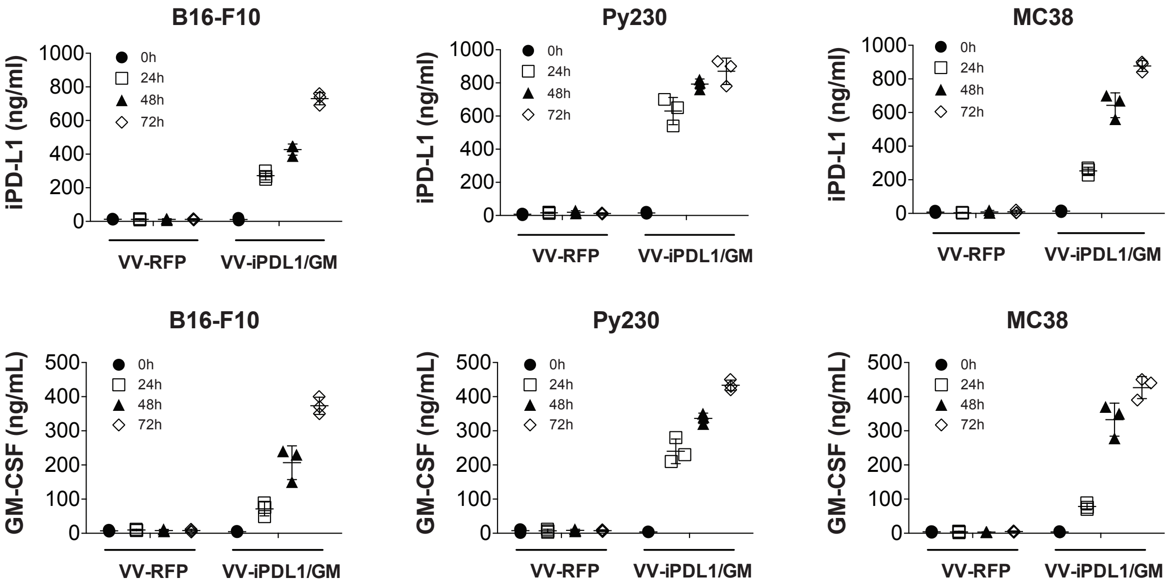

Supplementary Figure 1. Efficient secretion of GM-CSF and PD-L1 inhibitors (iPDL1) from VV-iPDL1/GM-infected tumor cells. B16-F10, Py230, or MC38 tumor cells were infected with VV-RFP or VV-iPDL1/GM at an MOI=2. 24, 48, or 72 h after VV infection, supernatants were collected and clarified. iPDL1 (PD1-Fc) was measured using mouse PD1 DuoSet ELISA kit (R&D, DY1021) and GM-CSF measured using mouse GM-CSF ELISA kit (Biolegend, Cat # 432204). n = 3 independent samples. Data presented as the means ± SD. The experiment was repeated twice.

Supplementary Figure. 2

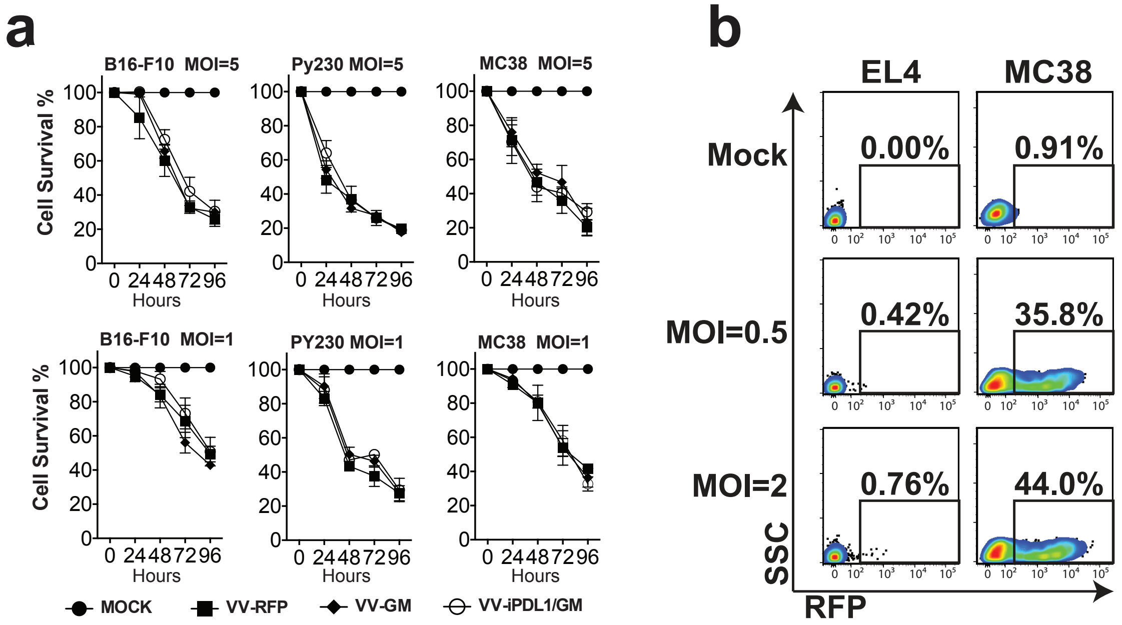

Supplementary Figure 2. Oncolytic activities of the recombinant virus VV-iPDL1/GM.

(a) Oncolytic activity of VVs against different tumor cells. Mouse tumor cells (B16-F10, Py230, MC38) were infected with the indicated VVs at an MOI of 1 or 5 for 24, 48, 72, and 96 h. MTT assay were performed to determine viability of infected tumor cells. The percentages of viable cells are presented at different time points. n = 3 independent samples. Data are presented as the means ± SD. Experiments were repeated twice.

(b) VV efficiently infected MC38 tumor cells, but not EL-4 tumor cells. Tumor cells seeded in 24-well plate were infected with the indicated MOI of VV-iPDL1/GM. 48 h later, the cells were harvested and analyzed by flow cytometry. RFP positive cells represent infected cells. Experiments were repeated three times.

Supplementary Figure. 3

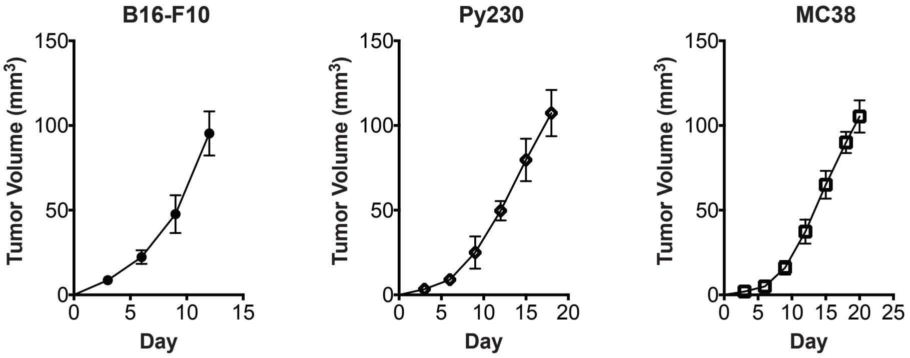

Supplementary Figure 3. Representative tumor growth rates prior to virus treatment. C57BL/6 mice were implanted with $5x10^5$ B16-F10, Py230, or MC38 cells subcutaneously. Tumor volume was monitored by caliper measurement on indicated days. n = 5 mice. Data presented as the means ± SD.

Supplementary Figure. 4

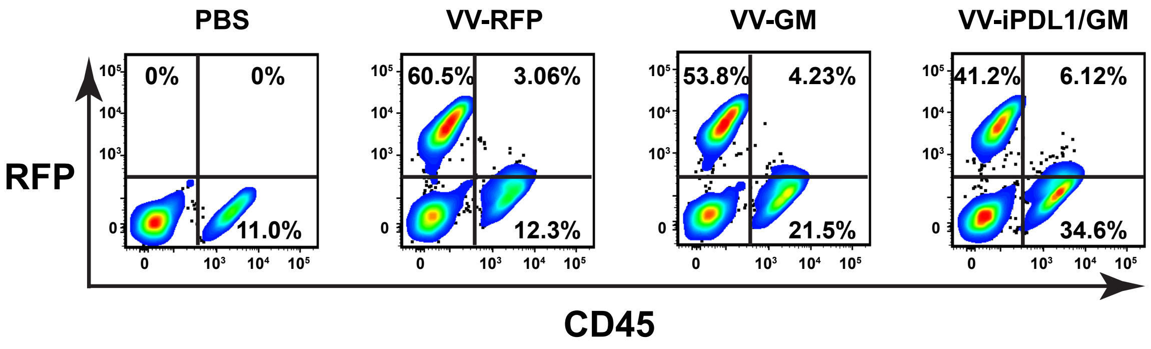

Supplementary Figure 4. Both CD45 positive and negative cells in tumor microenvironment were infected with intratumorally injected viruses. Groups of C57BL/6 mice were subcutaneously inoculated with MC38 cells ($1x10^6$). When tumor sizes reached ~100 mm^3 (counted as day 0), the tumors were intratumorally injected with 50 µL of PBS, VV-RFP, VV-GM or VV-iPDL1/GM (5 $x10^7$ pfu per tumor) on days 0 and 3. Two days post-2nd viral injection, VV-treated tumors were collected, weighed and digested with collagenase type I and DNase. A fraction of the tumor cell suspensions were blocked with anti-CD16/32 antibody and then stained with antibodies against CD45 to assess infected cells (RFP^+) frequency of CD45 positive and negative cells.

Supplementary Figure. 5

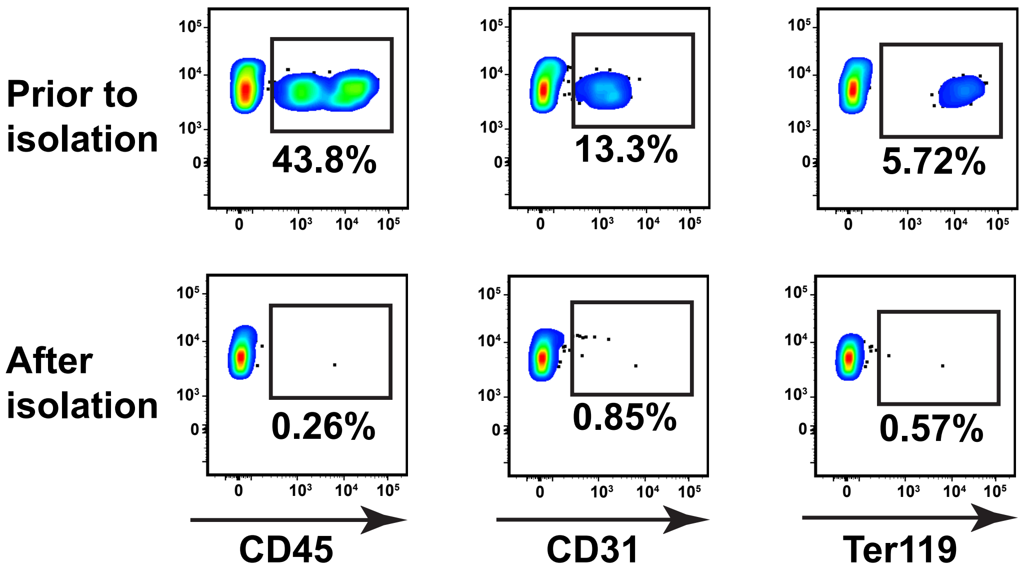

Supplementary Figure 5. Tumor purity was confirmed by flow cytometry after kit isolation. Using the same treatment schedule as in Supplementary Figure 4, tumor cells were isolated from the other fraction of the tumor cell suspensions using a tumor cell isolation kit (Miltenyl Biotec, Cat# 130-110-187). The isolated tumor cells and tumor cell suspensions before isolation were stained with lineage markers anti-CD45, CD31, and Ter119 antibodies**.**

Supplementary Figure. 6

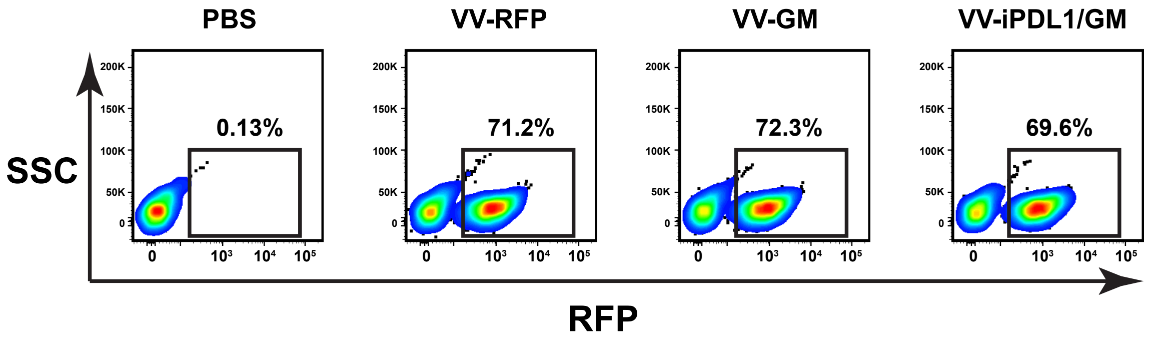

Supplementary Figure 6. Tumor cells were infected by injected viruses. Using the same treatment schedule as in Supplementary Figure 5, VV-infected cells (RFP^+) frequencies of isolated tumor cells were analyzed by flow cytometry.

Supplementary Figure. 7

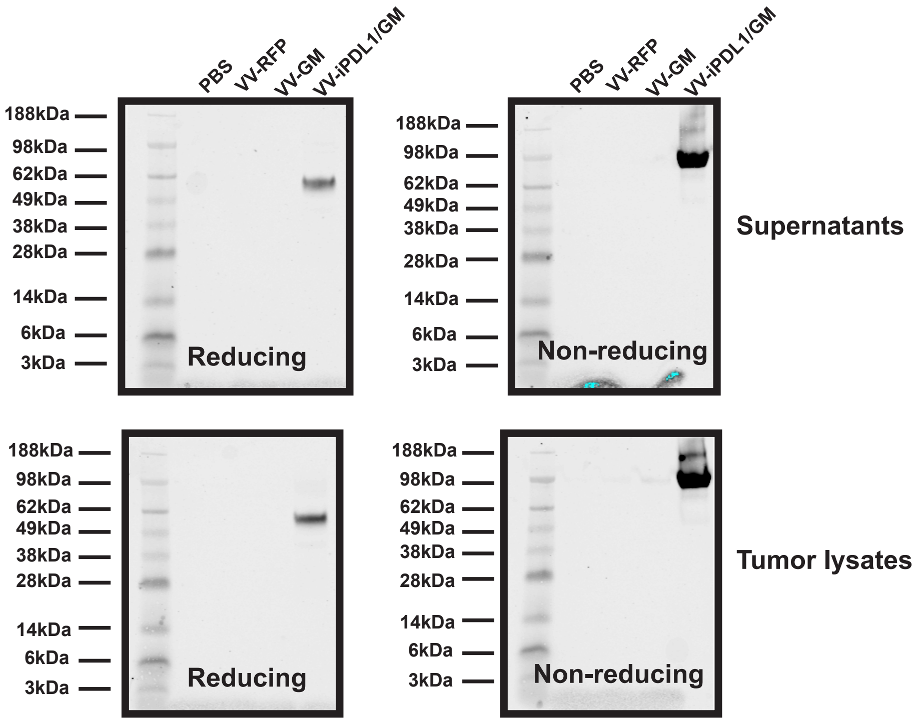

Supplementary Figure 7. Infected tumor cells were able to secrete iPDL1. Using the same treatment schedule as in Supplementary Figure 5, isolated tumor cells were further cultured *in vitro* for 48 h. Supernatants of the culture media and tumor cell lysates were analyzed by Western Blot using an anti-IgG Fc (Licor 926-32210) with reducing or non-reducing loading buffer. The experiment was repeated twice.

Supplementary Figure. 8

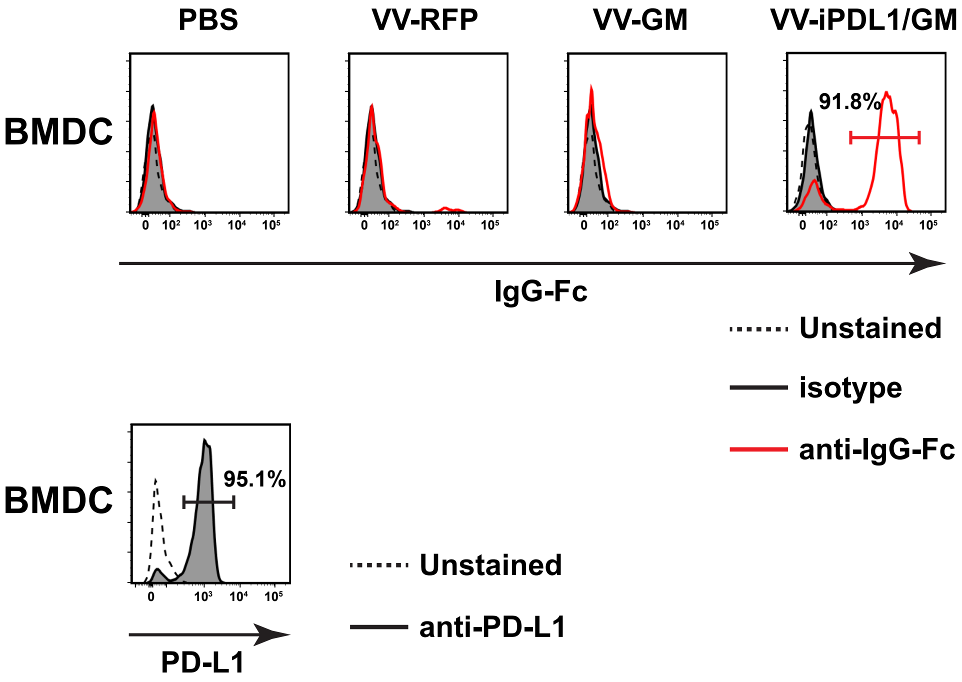

Supplementary Figure 8. **Secreted iPDL1 from infected isolated tumor cells binds to immune cells.** Mature bone marrow-derived dendritic cells (BMDC) were pre-treated with anti-CD16/32 antibodies to block FcRs binding on ice for 30 min. Cells were then incubated with the supernatants of the tumor cell culture media in Supplementary Figure 7, followed by stained anti-IgG-Fc or isotype IgG control. Mature BM-DC were also separately stained with anti-PD-L1.

Supplementary Figure. 9

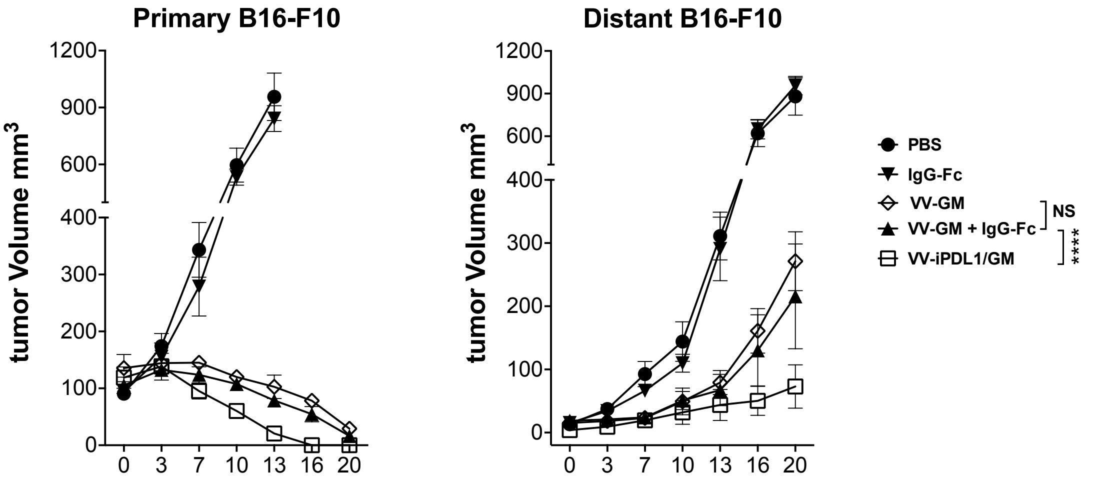

Supplementary Figure 9. Comparison of antitumor activities of VV-iPDL1 with the co-administrations of VV-GM and IgG Fc. B16-F10 melanoma cells were implanted to the left and right flanks of C57B/6 mice (5×10^5 to the left flank and 1×10^5 to the right flank). When the volume of left flank tumors reached ~100 mm^3 (counted as day 0), the mice were intratumorally injected with 50 μL of IgG-Fc (100 μg/tumor, Thermo, 31205) only, VV-GM (5 x10^7 pfu/tumor), VV-GM (5 x 10^7 pfu/tumor) + IgG-Fc (100 μg/tumor) (premixed and injected together), VV-iPDL1/GM (5 x 10^7 pfu/tumor), or PBS three times on days 0, 3, and 7. The left (treated) and right (untreated) flank tumor sizes were measured every 3-5 days. n= 5 mice. *P<0.05 by repeated-measures 2-way ANOVA. The experiment was repeated once.

Supplementary Figure. 10

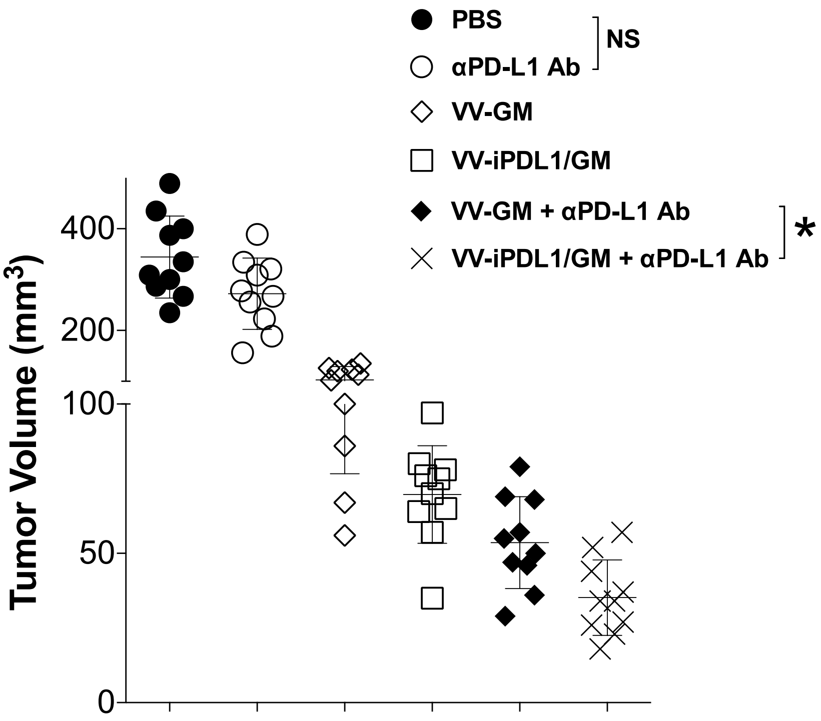

Supplementary Figure 10. Distribution of tumor volumes on day 10 after virus injection. Similar with B16-F10 tumor establishment in Figure **3a**, the mice were intratumorally injected with 50 μL of PBS, VV-GM, VV-iPDL1/GM (5x10^7 pfu per tumor) and 200 μg/mouse anti-PDL1 antibody intravenously each time on day 0, 3, and 7. n= 10 mice. Bars represent mean values ± SD. **P*<0.05 by two-tailed Mann–Whitney U test. The experiment was repeated once.

Supplementary Figure.11

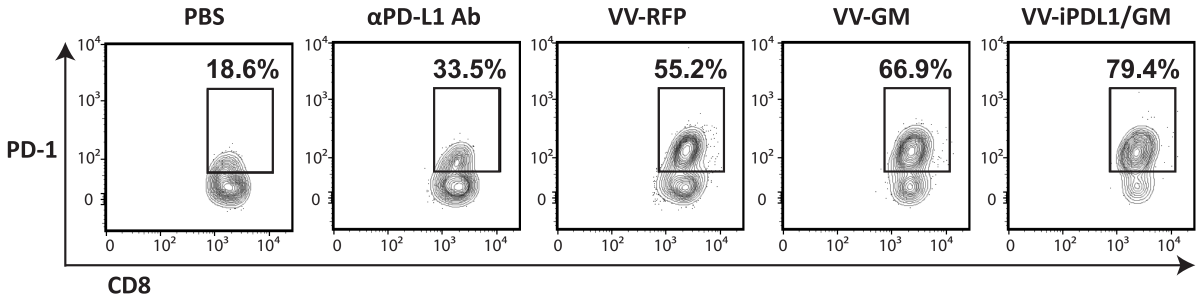

Supplementary Figure 11. PD-1 expression of CD8+ T cell in virus-treated tumors. A similar treatment schedule as in Figure 5a was used, VV-treated MC38 tumors were harvested, weighed and digested for preparation of single cell suspensions followed by antibody staining against CD45, CD3, CD8, or PD-1.

Supplementary Figure. 12

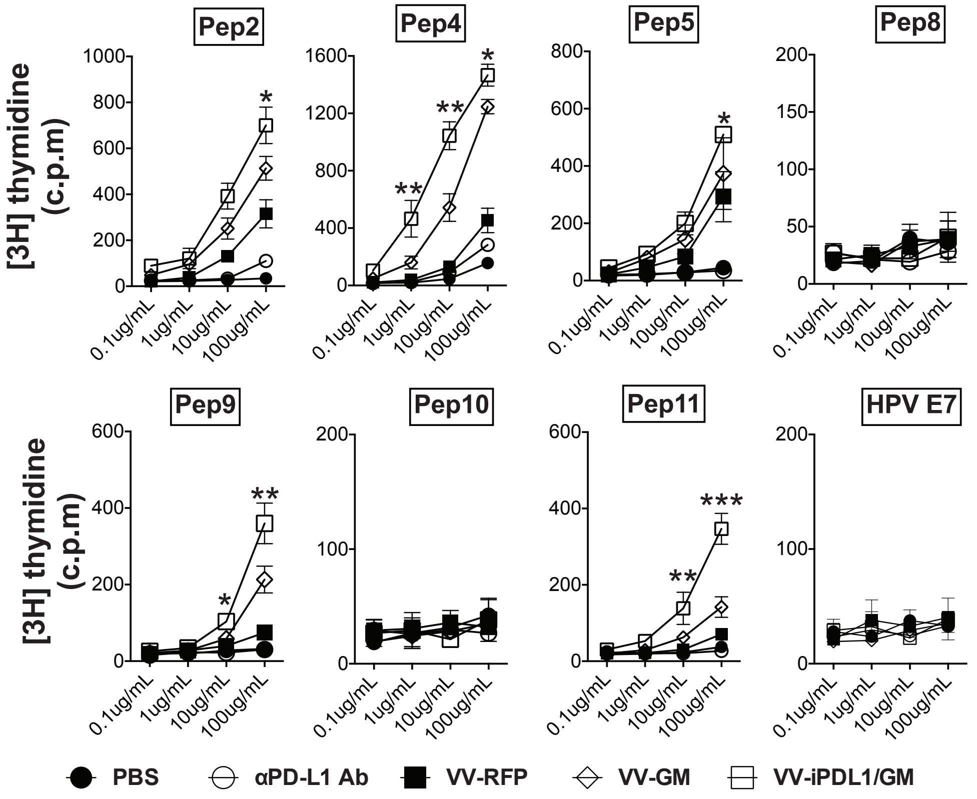

Supplementary Figure. 12. Enhanced T cell responses against various neoantigen peptides. MC38 tumor-bearing mice were intratumorally injected with PBS or various VVs as described in **Figure 6a**. The splenocytes from the VV-treated tumor-bearing mice were cultured in complete RPMI1640 in 96 well plates ($1x10^5$ per well) in the presence of one of indicated neoepitope peptides at various concentrations for 80 h. [^{3}H]thymidine incorporation was measured. n = 3 mice. Data presented as the means ± SD. **P*<0.05, ***P*<0.01 by two-tailed Student's *t*-test.

Supplementary Figure. 13

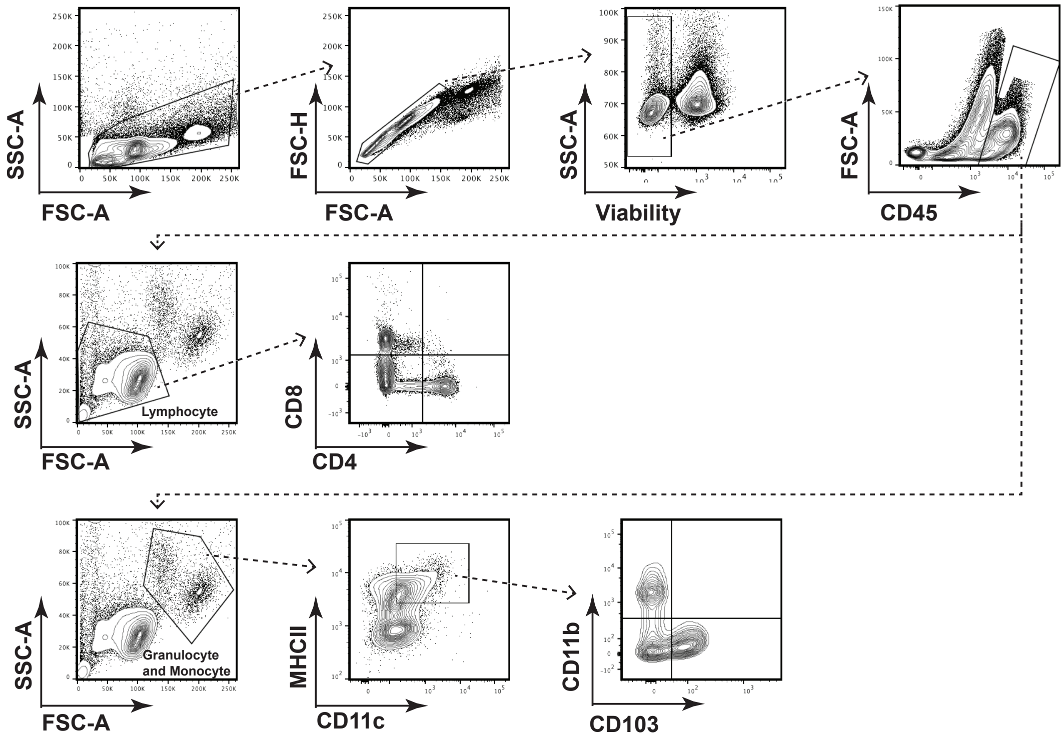

Supplementary Figure. 13. Flow cytometric analysis of tumor-infiltrating cells. Using the same treatment schedule as in **Figure. 5a**, tumor cell suspensions from VV treated mice were analysed by FACS. Gate strategy of $CD4^+$ or $CD8^+$ lymphocytes, and $CD103^+$ DCs.

Supplementary Figure. 14

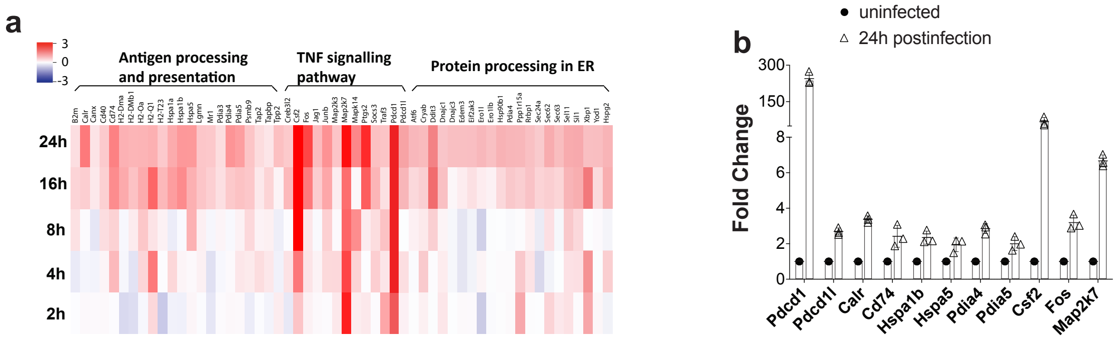

Supplementary Figure 14. Altered gene expression of VV-iPDL1/GM-infected MC38 tumor cells. (a) RNA-seq analysis of VV-iPDL1/GM-infected MC38 cells. MC38 cells were infected with VV-iPDL1/GM for the indicated times. The infected tumor cells were harvested and the extracted total RNA was used for RNA-Seq. **(b) qRT-PCR of 11 identified genes**. The gene expression level was quantified and normalized to the GADPH control. Bars depict SD (n=3 independent samples).

Supplementary Figure 15

A. DNA sequence of iPDL1 fusion gene

5’-
tttacccggagtccgggagaagctcttagtcgtgtggtgattgtgcagaccctcgtggaccactgaacaggagtagctatttctttccaccca
gttcttcttttccactctcagcttgctgtacatgaagtaagaaccatcagagtccaggactggttcagtgttcttgtagtttagctctgttttcccgtt
gttggtccactccacgtaaatgtcttcaggcatgaagtctgtgaccatgcaggtcagagtgacctgtttcttagtcatctcttcttctggtggagg
caagacatatacctgtggagctcttactgaccctttgggttttgagatggttctctcgatgggcgctgggaggtctttgttgttgaccttgcatttg
aactccttgccactcatccagtcctggtgctggatggggagggcactgaccacccggagagtactgttgtaatcctctctatgggtttgtgtct
gagctgtgtgtacttccacgttgttcacaaaccagctgatctggacatctgggtcatcctcgctcacatccaccaccacacatgtgactatggg
gctcagggagatcatgagtacatccttgatctttggagggaagatgaagacggatggtccacccaagaggttaggtgctgggcatttgcatg
gaggacagggcttgattgtgggccctctgggttgaaaccggccttctggtttgggcgaggggctgggatatcttgttgaggtctccaggattc
tctctgttaccacgagctctgctccagggctctcctcgatttttgccttggggtgcagggagatggccccacagaggtagatgccactgtcatt
gcgccgtgtgtcaaggatgttcatgtggaagtcatgcctgttgggcagctgtatgatctggaagcgggcatcctggacgggttggctcaaac
cattacagaaggcggcctgtttttcagtctggttgctgggactcaggcggttccagttcagcataagatcctccgaccagttggacaagctgc
aggtgaaggtggcatttgctccctctgacactgtgagccaggctgggtagaaggtgagggacctccagggcccattggggacctctagaa
gccaccctgattgccagctcaactgcagcacagcccaagtgaatgaccagggtacctgccggacccacat-3’

B. Amino acid sequence of iPDL1

N’-

MWVRQVPWSFTWAVLQLSWQSGWLLEVPNGPWRSLTFYPAWLTVSEGANATFTCSLS

NWSEDLMLNWNRLSPSNQTEKQAAFCNGLSQPVQDARFQIIQLPNRHDFHMNILDTRR

NDSGIYLCGAISLHPKAKIEESPGAELVVTERILETSTRYPSPSPKPEGRFQPRGPTIKPCPP

CKCPAPNLLGGPSVFIFPPKIKDVLMISLSPIVTCVVVDVSEDDPDVQISWFVNNVEVHTA

QTQTHREDYNSTLRVVSALPIQHQDWMSGKEFKCKVNNKDLPAPIERTISKPKGSVRAP

QVYVLPPPEEEMTKKQVTLTCMVTDFMPEDIYVEWTNNGKTELNYKNTEPVLDSDGSY

FMYSKLRVEKKNWVERNSYSCSVVHEGLHNHHTTKSFSRTPGK*

Supplementary table I

Name	Peptide	MHC allele	IC_{50}(mutant)	IC_{50}(Wild Type)
Pep1	AALLNSA(G/**V**)L	H-2D^b	3nM	52nM
Pep2	AQL(P/**A**)NDVVL	H-2D^b	9nM	100nM
Pep3	MAPIDHT(A/**T**)M	H-2D^b	30nM	102nM
Pep4	ASMTN(R/**M**)ELM	H-2D^b	2nM	3nM
Pep5	SIIVFNL(V/**L**)	H-2K^b	8nM	34nM
Pep6	SSP(D/**Y**)SLHYL	H-2D^b	211nM	685nM
Pep7	(S/**I**)MTQHLEPI	H-2D^b	78nM	29nM
Pep8	SAIRSYQ(D/**Y**)V	H-2D^b	35nM	755nM
Pep9	VSPVND(V/**L**)DV	H-2D^b	44nM	18nM
Pep10	MG(G/**V**)MNRRPI	H-2D^b	77nM	841nM
Pep11	FM(A/**S**)CNLLLV	H-2D^b	79nM	24nM
HPV E7	YMLDLQPETT	H-2D^b	Irrelevant peptide	
$OVA_{(257-264)}$	SIINFEKL	H-2K^b	Irrelevant peptide	

Supplementary Table I. Neoantigenic epitope peptides used in this study.

Supplementary Table 2

18S	5'- cggctaccacatccaaggaa-3'	3'- gctggaattaccgcggct-5'
Cxcl10	5'- gctgccgtcattttctgc-3'	3'- tctcactggcccgtcatc-5'
Calr	5'- aaaggaccctgatgctgccaag-3'	3'- tgttcggtctcgtgtagggact-5'
Cd74	5'- gctggatgaagcagtggctctt-3'	3'- ggtccttcttcagtcggtgtag-5'
Hspa1b	5'- acaagtcggagaacgtgcagga-3'	3'- gaagtggtggatgagcctgttg-5'
Hspa5	5'- tgtcttctcagcatcaagcaagg-3'	3'-ttcggacaggtccttcacaacc-5'
Pdia4	5'- gaccagtttgtgaaggagcactc-3'	3'- acttcaggaggtgcctctacga-5'
Csf2	5'- aacctcctggatgacatgcctg-3'	3'- tcgtcccagatgccccgttaaa-5'
Fos	5'- gggaatggtgaagaccgtgtca-3'	3'- cccttgccttattctaccgacg-5'
Map2k7	5'- tcaggtgtggaagatgcggttc-3'	3'- gagttctcggtactgacgggaa-5'
Pdcd1	5'- cggtttcaaggcatggtcattgg-3'	3'- ccttcgttcctgctgtgagact-5'
Pdcd1l	5'- tgcggactacaagcgaatcacg-3'	3'- gctcccaataggtcttcgactc-5'

Supplementary Table 2. List of primer sequences.
