# Supplementary material for: An engineered oncolytic virus expressing PD-L1 inhibitors activates tumor neoantigen-specific T cell responses
Source: Nat Commun. 2020 Mar 13;11:1395. doi: 10.1038/s41467-020-15229-5 (PMC7070065; doi:10.1038/s41467-020-15229-5)
Supplement: Supplementary file 2 — Reporting summary [file 41467_2020_15229_MOESM2_ESM.pdf]

nature research

Corresponding author(s): Si-Yi Chen & Xue F Huang

Last updated by author(s): Jan 19, 2020

Reporting Summary

Nature Research wishes to improve the reproducibility of the work that we publish. This form provides structure for consistency and transparency in reporting. For further information on Nature Research policies, see Authors & Referees and the Editorial Policy Checklist .

Statistics

For all statistical analyses, confirm that the following items are present in the figure legend, table legend, main text, or Methods section.

n/a	Confirmed	
☐	☒	The exact sample size (*n*) for each experimental group/condition, given as a discrete number and unit of measurement
☐	☒	A statement on whether measurements were taken from distinct samples or whether the same sample was measured repeatedly
☐	☒	The statistical test(s) used AND whether they are one- or two-sided<br>*Only common tests should be described solely by name; describe more complex techniques in the Methods section.*
☒	☐	A description of all covariates tested
☐	☒	A description of any assumptions or corrections, such as tests of normality and adjustment for multiple comparisons
☐	☒	A full description of the statistical parameters including central tendency (e.g. means) or other basic estimates (e.g. regression coefficient) AND variation (e.g. standard deviation) or associated estimates of uncertainty (e.g. confidence intervals)
☐	☒	For null hypothesis testing, the test statistic (e.g. *F*, *t*, *r*) with confidence intervals, effect sizes, degrees of freedom and *P* value noted<br>*Give P values as exact values whenever suitable.*
☒	☐	For Bayesian analysis, information on the choice of priors and Markov chain Monte Carlo settings
☒	☐	For hierarchical and complex designs, identification of the appropriate level for tests and full reporting of outcomes
☒	☐	Estimates of effect sizes (e.g. Cohen's *d*, Pearson's *r*), indicating how they were calculated

Our web collection on statistics for biologists contains articles on many of the points above.

Software and code

Policy information about availability of computer code

Data collection	odyssey v3.0, MikroWin2000, Living Image v4.4, FACS DIVA 6.1.2,
Data analysis	Illustrator CS6, flowjo 10.4.0, Graphpad prism 6 , Microsoft excel 2011 for mac, Living Image v4.3.1, RNAseq analysis was performed with the following software HTSeq v0.5.3,Solexa pipeline v1.8, FastQC software 0.11.7, Hisat2 software, StringTie 1.3.3, R 3.4.1, Python 2.7.

For manuscripts utilizing custom algorithms or software that are central to the research but not yet described in published literature, software must be made available to editors/reviewers. We strongly encourage code deposition in a community repository (e.g. GitHub). See the Nature Research guidelines for submitting code & software for further information.

Data

Policy information about availability of data

All manuscripts must include a data availability statement. This statement should provide the following information, where applicable:

- Accession codes, unique identifiers, or web links for publicly available datasets
- A list of figures that have associated raw data
- A description of any restrictions on data availability

All data generated or analysed during this study are included in this published article (and its supplementary information files), and are available from the corresponding author on reasonable request

Field-specific reporting

Please select the one below that is the best fit for your research. If you are not sure, read the appropriate sections before making your selection.

Life sciences study design

All studies must disclose on these points even when the disclosure is negative.

Sample size	Group size was based on previous experience. For example, Experiments were performed in biological triplicates n=3 unless otherwise noted. And sample size of at least 5 mice in each group was widely accepted in life science experiment to support the consistency of the data and conclusion. No statistical method was used to predetermine sample size
Data exclusions	No data was excluded. In Figure 3, when tumors reach >1000 mm^3, the mice were euthanized in accordance with the guidelines of the Institutional Animal Care and Use Committee of USC.
Replication	All attempts at replication were successful, and standard deviations were within expected ranges. Unless otherwise noted, each experiment was repeated three or more times.
Randomization	After tumor injection when tumor size reached about 100mm^3, all mice were allocated randomly for each group. And within animal controls were performed wherever possible. And the possible confounders 'experimenter' and 'day of experiment' were equally matched between groups. For experiments other than mice studies, randomization is irrelevant to our study, because different groups were defined by either different cell lines or different conditions.
Blinding	For all in vitro or vivo experiments whose data were generated by machine reading, blinding or not won't affect the results. For caliper measurement of tumor size, different people were involved in measuring even they were aware of group labeling which was conducted partially blinded.

Reporting for specific materials, systems and methods

We require information from authors about some types of materials, experimental systems and methods used in many studies. Here, indicate whether each material, system or method listed is relevant to your study. If you are not sure if a list item applies to your research, read the appropriate section before selecting a response.

Materials & experimental systems

n/a	Involved in the study	
☐	☒	Antibodies
☐	☒	Eukaryotic cell lines
☒	☐	Palaeontology
☐	☒	Animals and other organisms
☒	☐	Human research participants
☒	☐	Clinical data

Methods

n/a	Involved in the study	
☒	☐	ChIP-seq
☐	☒	Flow cytometry
☒	☐	MRI-based neuroimaging

Antibodies

Antibodies used	anti-CD16/32 (clone: 93, Cat: 101319, Lot: B247951, Biolegend, 1:100), anti-PD-L1 (PE-cy7, clone: 10F.9G2, Cat: 124313, Lot: B249444, Biolegend; Clone: MHI5, eBioscience, 1:100), anti-IgG2a-Fc (APC, Cat: 31983, Lot: TL2686983, Thermo, 1:500), anti-CD45 (clone: 30-F11. BV421, Cat: 103133, Lot: B229121, Biolegend, or PE, Cat: 12-0451-82, Lot: E013632, eBioscience, 1:500), anti-CD11c (clone: HL3, PE, Cat: 553802, Lot: 50586, BD Biosciences; or APC, Cat: 117310, Lot: B166840, Biolegend, 1:100), anti-CD11b (clone: M1/70, eF450, Cat: 48-0112-82, Lot:E10253-1631, eBioscience; or PE-cy5, Cat: 101209, Lot: B260952, Biolegend, 1:100), anti-CD103 (FITC, clone: 2E7, Cat: 121419, Lot: B213630, Biolegend, 1:100), viability dye (BV510, REF: 13-0870-T100, Lot: D0870061416133 or UV450, REF: 13-0868-T100, Lot; D0868010416133, Tonbo Biosciences, 1:1000), anti-CD3 (Pacific Blue, clone: 17A2, Biolegend, Cat: 100203, 1:1000), anti-CD4 (clone: RM4-5, PE, Cat: 553730, Lot: 47561, BD Biosciences, or PE-cy5, REF: 15-0041-82, Lot: E06071-1631, eBioscience, 1:500), anti-CD8 (FITC, Cat:100705, Lot: B217241; APC, Cat: 100711, Lot: B22411, or APC-cy7, Cat: 100713, Lot: B237525, clone: 53-6.7, Biolegend, 1:1000), anti-Gr-1 (PE, Cat: 553128, Lot: 29290; APC-cy7, Cat: 557661, Lot: 55732, BD biosciences, clone: RB6-8C5, Biolegend, 1:100), MHCII (clone: M5/114.15.2, FITC, Cat: REF: 11-5322-81, Lot: 4287995, eBioscience or PE, Cat: 557000, Lot: 60577, BD Biosciences, 1:100),<br>anti-FoxP3 (PE, Cat: 12-5773-80, Lot: E01763-1631 or FITC, Cat: 11-5773-82, Lot: E031743, clone: FJK-16s, eBioscience, 1:100), anti-IFN-γ (APC, clone: XMG1.2, Cat: 554413, Lot: 73070, BD biosciences, 1:100), anti-107a (PE-cy7, Cat: 560647, Lot: 6326658, clone: 1D4B, BD biosciences, 1:500), anti-TNFα (PE, clone: MP6-XT22, Cat: 554419, Lot: 4059871, BD Biosciences, 1:100), anti-IL-2 (PE, Cat: 561061, Lot: 5295812, clone: JES6-5H4, BD Biosciences, 1:100), anti-IL-12 (PE-cy7, Cat:505209, clone: C15.6, Biolegend, 1:100), anti-CD80 (FITC, Cat: 553768, Lot: 09530, clone: 16-10A1, BD Biosciences, 1:100), anti-CD86 (APC, Cat: 558703, Lot: 33261, clone: GL-1, BD Biosciences, 1:100), and anti-CD40 (PE, Cat: 553791, Lot: 0000066846, clone 3/23, BD Biosciences, 1:100). Pentamer H-2Db-ASMTNMELM-PE was provided by ProImmune Inc (F2695-2A-D) (1:100). Anti-CD40 (Cat: BP0016-2, Lot: 671717N1, BioXcell). Anti-PD-L1(clone: 10F.9G2, Cat: BE0101, Lot: 665717O1B, BioXcell). anti-CD31 (FITC, clone: 390, Cat: 102405, Biolegend, 1:100). anti-Ter119 (APC, clone: TER-119, Cat: 116211, Biolegend, 1:100). IRDye® 800CW Goat anti-Mouse IgG Secondary Antibody (Polyclonal, Cat: 926-32210, Lot: C50909-01,). Anti-PD-1 (Clone: RMP1-30, Cat: 101319, Lot: B247951)
Validation	Anti-CD40 (Cat: BP0016-2, Lot: 671717N1, BioXcell). Anti-PD-L1(clone: 10F.9G2, Cat: BE0101, Lot: 665717O1B, BioXcell),IRDye® 800CW Goat anti-Mouse IgG Secondary Antibody Antibodies were validated by the manufacturer western Blot assay. All the

other antibodies were validated by flow cytometry.

Eukaryotic cell lines

Policy information about cell lines

Cell line source(s)

Human embryonic kidney cell line 293T, osteosarcoma HUTK-143B, monkey kidney fibroblasts CV1, murine adenocarcinoma Py230, murine melanoma B16-F10, Murine lymphoma EL4 were purchased from the American Type Culture Collection (ATCC). Murine colon adenocarcinoma cells MC38 was purchased from Kerafast

Authentication

Certificates of analysis were provided with cell line by ATCC. ATCC authenticates cell lines routinely with the following tests:
Short Tandem Repeat (STR) Profiling
Cellular Morphology
Karyotyping
Cytochrome C Oxidase I (COI) Assay Testing

Mycoplasma contamination

All cell lines were routinely tested for mycoplasma contamination and found negative.

Commonly misidentified lines
(See ICLAC register)

None

Animals and other organisms

Policy information about studies involving animals; ARRIVE guidelines recommended for reporting animal research

Laboratory animals

C57BL/6 mouse, female, 7-8 weeks.

Wild animals

the study didn't involve wile animals

Field-collected samples

The study didn't involve samples collected from the field

Ethics oversight

Institutional Animal Care and Use Committee of USC which IACUC approved

Note that full information on the approval of the study protocol must also be provided in the manuscript.

Flow Cytometry

Plots

Confirm that:

☐ The axis labels state the marker and fluorochrome used (e.g. CD4-FITC).

☒ The axis scales are clearly visible. Include numbers along axes only for bottom left plot of group (a 'group' is an analysis of identical markers).

☐ All plots are contour plots with outliers or pseudocolor plots.

☒ A numerical value for number of cells or percentage (with statistics) is provided.

Methodology

Sample preparation

For Figure 1d. Isolation of bone marrow cells – Age-matched mice were sacrificed by cervical dislocation and bones from hind legs, forelegs were isolated. Bone marrow (BM) was extracted by crushing the bones using mortar and pestle in RPMI + 2% FBS. The suspension was filtered through a 40 µm mesh and centrifuged for 5 min at 1,500 rpm. Freshly isolated bone marrow cells from mice were cultured in complete RPMI1640 media supplemented with 10% FBS, 20 ng/mL GM-CSF, and 40 ng/mL IL-4 for dendritic cell differentiation.
For Figure 4.
Mice with tumors are euthanized by CO2 asphyxiation. Using autoclaved surgical instruments rinsed with 70% ETOH, cut tumor into small (< 3mm) pieces and incubate in 5 ml dissociation solution (RPMI medium supplemented with 5% FBS, Collagenase type I (200 U/ml) and DNase I (100 µg/ml)) for 30 min at 37 °C, Pipetting (using a 1,000 µl pipet tip) and vortexing every 10 minutes during the incubation. If myeloid cells will be subsequently isolated, substitute 5% FBS and Collagenase type I with 10% FBS and Collagenase type IV (200 U/ml), respectively. Splenic cells were obtained by physical dissociation of the spleen on a cell strainer. After incubation, pass cell suspension through a 70 µm cell strainer and wash twice with 10 ml PBS buffer for staining. Cells were blocked with Fc blocking reagent, and then stained with antibody cocktail for 30 min on ice. If intracellular staining was performed, BD Cytofix/Cytoperm Fixation and Permeabilization Solution was used according to the manufacturer's manual. Counting beads (San diego, biolegend) were added in the final cell suspension to quantify cells when necessary.

Instrument

BD canto II, BD ariaIII

Software

Collection: FACS DIVA
analysis: Flowjo 10.4.0

Cell population abundance	Purity of post-sort fractions is regularly measured by flow for CD8+ and purity was >95%
Gating strategy	Starting cells were gated on a linear FSC/SSC plot, and then negative population in viability stain lower than 10^3 was selected as live cells for the following gating which was shown in all flow figures. Positive/negative populations were determined by FMO controls

☒ Tick this box to confirm that a figure exemplifying the gating strategy is provided in the Supplementary Information.
